# Supplementary material for: Global mapping of protein–metabolite interactions in Saccharomyces cerevisiae reveals that Ser-Leu dipeptide regulates phosphoglycerate kinase activity
Source: Commun Biol. 2021 Feb 10;4:181. doi: 10.1038/s42003-021-01684-3 (PMC7876005; doi:10.1038/s42003-021-01684-3)
Supplement: Supplementary file 2 — Supplementary Material [file 42003_2021_1684_MOESM2_ESM.pdf]

Supplementary Information

Global mapping of protein-metabolite interactions in *Saccharomyces cerevisiae* reveals that Ser-Leu dipeptide regulates phosphoglycerate kinase activity.

Marcin Luzarowski,[1*] Rubén Vicente,[2] Andrei Kiselev,[1,3] Mateusz Wagner,[1,4] Dennis Schlossarek,[1] Alexander Erban,[1] Leonardo Perez de Souza,[1] Dorothee Childs,[5] Izabela Wojciechowska,[1] Urszula Luzarowska,[1,6] Michał Górka,[1] Ewelina M. Sokołowska,[1] Monika Kosmacz,[1] Juan C. Moreno,[1] Aleksandra Brzezińska,[1] Bhavana Vegesna,[1] Joachim Kopka,[1] Alisdair R. Fernie,[1] Lothar Willmitzer, [1] Jennifer Ewald,[7] Aleksandra Skirycz[1,8*]

[1] Max Planck Institute of Molecular Plant Physiology, Department of Molecular Physiology, Am Mühlenberg 1, 14476 Potsdam, Germany
[2] Max Planck Institute of Molecular Plant Physiology, Department of Metabolic Networks, Am Mühlenberg 1, 14476 Potsdam, Germany
[3] Laboratoire de Recherche en Sciences Végétales (LRSV), UPS/CNRS, UMR 5546, Castanet Tolosan, France
[4] University of Wrocław, Faculty of Biotechnology, Laboratory of Medical Biology, F. Joliot-Curie 14A, 50-383 Wrocław, Poland
[5] European Molecular Biology Laboratory, Department of Genome Biology, Meyerhofstraße 1, 69117 Heidelberg, Germany
[6] Ben-Gurion University of the Negev, Department of Life Sciences, Beer-Sheva 8410501 Israel
[7] Interfaculty Institute of Cell Biology, Eberhard Karls University of Tuebingen, Auf der Morgenstelle 15, 72076 Tuebingen, Germany
[8] Boyce Thompson Institute, 533 Tower Rd, 14853 Ithaca, NY, US

[*]Correspondence skirycz@mpimp-golm.mpg.de, luzarowski@mpimp-golm.mpg.de

Supplementary Tables

Supplementary Table S1. A list of materials used in this study.

Reagent/Resource	Reference/Source	Identifier/Catalogue number
	Experimental strains	
YSBN2	[1]	Y40383
Yeast ORF collection	Dharmacon [2]	YSC3870
S288c	ATCC	204508
	Chemicals, enzymes and reagents	
YPD BROTH	Sigma-Aldrich	Y1375
Ammonium bicarbonate	Sigma-Aldrich	9830
NaCl	Sigma-Aldrich	S7653
MgCl2	Sigma-Aldrich	2189.1
DTT	Sigma-Aldrich	D0632
PMSF	Sigma-Aldrich	P7626
NaF	Sigma-Aldrich	S6776
Na3VO4	Sigma-Aldrich	S6580
Protease inhibitor cocktail	Sigma-Aldrich	P9599
MTBE	Biosolve	138906
Methanol	Biosolve	136806
Water	Biosolve	232106
Acetonitrile	Biosolve	12006
Trifluoroacetic acid	Biosolve	202341
Formic acid	Biosolve	69141
Bradford reagent	Sigma-Aldrich	B6916
Urea	Sigma-Aldrich	U5128
Thiourea	Sigma-Aldrich	T8656
Iodoacetamide	Sigma-Aldrich	I1149
Trypsin/Lys-C Mix, Mass Spec Grade	Promega	V5071
C18 Empore® extraction discs	Fisher Scientific	2215
SD-Ura Broth	Takara	630314

Yeast synthetic drop-out medium without Uracil	Sigma-Aldrich	Y1501
Yeast nitrogen base without amino acids	Sigma-Aldrich	Y0626
Raffinose	Sigma-Aldrich	R0250
Yeast extract	Sigma-Aldrich	Y1625
Bacto-peptone	Sigma-Aldrich	91249
Galactose	Sigma-Aldrich	G0750
Tris base	Sigma-Aldrich	10708976001
cOmplete EDTA-free protease inhibitor cocktail	Merck	11873580001
EDTA	Sigma-Aldrich	3609
TEMED	Carl Roth	2367.3
Rotiphorese Gel 30 (37.5:1)	Carl Roth	3029.2
Silica/zirconia beads	Biospec	11079105z
IgG Sepharose® 6 Fast Flow beads	GE-Healthcare	17-0969-01
Mobicol "Classic" filter	MoBiTec	M1002S and M513515
Protease 3C	Thermo Fisher Scientific	88947
Glutathione agarose	Sigma-Aldrich	G4510
10 kDa MWCO Amicon Filter	Sigma-Aldrich	Z706345
Pgk1	Sigma-Aldrich	P7634
RED-MALEIMIDE labelling kit	Nanotemper	MO-L004
Tween 20	Nanotemper	Part of the labeling kit
Inosine	Sigma-Aldrich	I4125
Hypoxanthine	Sigma-Aldrich	H9371
Xanthine	Sigma-Aldrich	X7375
Ser-Leu	Biomatik	Custom order
Tyr-Asp	Biomatik	Custom order
Ser-Leu Agarose Beads	Cube Biotech	Custom order
Standard Capillaries MST	Nanotemper	MO-K022
TCEP	Sigma-Aldrich	C4706
Serine	Sigma-Aldrich	S4500

Standard Capillaries Prometheus	Nanotemper	PR-C002
G3P	Sigma-Aldrich	G7886
GAPDH	Sigma-Aldrich	G2267
TPI	Sigma-Aldrich	T2391
GDH	Roche	10127752001
GPOX	Roche	Custom order
ATP	Roche	10127531001
3PGA	Sigma-Aldrich	P8877
NADH	Roche	10128023001
Triton X-100	Sigma-Aldrich	X100
KCl	Carl Roth	6781.1
PageRuler Prestained Protein Ladder	Thermo Fisher Scientific	26616
Equipment		
Mixer Mill MM 400	Retsch GMbH	207450001
Sepax SRT-10 SEC-300 21.2 × 300 mm column	Sepax Technologies	225300-21230
ÄKTA explorer 10	GE Healthcare Life Science	18130000
Q Exactive HF Hybrid Quadrupole-Orbitrap Mass Spectrometer	Thermo Fisher Scientific	IQLAAEGAAPFALGMBFZ
ACQUITY UPLC M-Class system	Waters	NA
Monolith NT.115	Nanotemper	NA
Prometheus NT.48	Nanotemper	NA
Q Exactive Plus Hybrid Quadrupole-Orbitrap Mass Spectrometer	Thermo Fisher Scientific	IQLAAEGAAPFALGMBDK
Software		
Expressionist Refiner MS 11.0	Genedata	NA
MaxQuant	[3,4]	NA
R	[5,6]	NA
Xcalibur	Thermo Fisher Scientific	NA

MO Affinity Analysis	Nanotemper	NA
Adobe Illustrator CS5	Adobe	NA

Supplementary Methods

Yeast growth conditions, cell lysis, and extraction of native complexes for PROMIS.

The YSBN2 strain of *S. cerevisiae* was cultivated at 28 °C with moderate shaking until it reached the logarithmic phase (OD600 = 0.3–0.5). Cells were collected by centrifugation (4,000 g, 4 °C, 20 min), washed with AmBIC buffer (50 mM ammonium bicarbonate, 150 mM NaCl, 1.5 mM $MgCl_2$), and centrifuged again (4,000 g, 4 °C, 20 min). The yeast pellet was solubilised in a small volume of buffer and then snap frozen in liquid nitrogen. Cells were ground four times for one min using a Retsch mill at 30 RPS and stored at -80 °C until used. A 0.7 mL volume of lysis buffer (50 mM AmBIC, 150 mM NaCl, 1.5 mM MgCl2, 5 mM DTT, 1 mM PMSF, 1 × Protease Inhibitor Cocktail (Sigma-Aldrich), 0.1 mM Na3VO4, and 1 mM NaF) was added to1 g of ground yeast material, and the mixture was gently vortexed (with two metal balls) until it thawed. The mixture was centrifuged for 20 min at 4,000 g, 4 °C (Beckman Coulter), and the supernatant was transferred to ultracentrifuge tubes and centrifuged for one hour at 35,000 RPM (max. 148,862 g, avg. 116,140 g) at 4 °C to yield a soluble fraction containing endogenous complexes. The soluble fraction was loaded onto previously pre-rinsed (15 mL wash buffer: 50 mM AmBIC, 150 mM NaCl, 1.5 mM $MgCl_2$, 20 min, 4,000 g, 4 °C) Amicon Ultra-15 centrifugal filter units (10 kDa MWCO) and centrifuged for 20 min at 4,000 g, 4 °C. Experiments were conducted using three independently inoculated, grown, harvested, and extracted yeast cultures.

LC-MS/MS of proteins.

The Bradford assay (Carl Roth GmbH + Co. KG, Karlsruhe, Germany) was used to determine the protein concentration of SEC fractions. Protein pellets from fractions A6–C13 were resuspended in 30 µL urea buffer (6 M urea, 2 M thiourea in 40 mM ammonium bicarbonate). Reduction of cysteines, alkylation, and enzymatic digestion using LysC/Trypsin Mix (Promega Corp., Fitchburg, WI) were performed on 20 µg of proteins from each fraction according to the manufacturer’s instructions. Digested proteins were desalted on self-made C18 Empore® extraction discs (3M, Maplewood, MN) STAGE tips [7], concentrated using the centrifugal evaporator to approximately 4 µL and stored at -80 °C until measured.

Dried peptides were resuspended in 60 µl MS loading buffer (2% ACN, 0.2 % TFA), and 3 µl (equivalent to 0.8–1.0 µg of peptides) were separated using C18 reversed-phase column connected to an ACQUITY UPLC M-Class system in a 120 min gradient. The gradient started from 3.2% and increased to 7.2% ACN in 20 min, to 24.8% ACN over 70 min and to 35.2% ACN over 30 min, followed by a 5 min washout with 76% ACN.

The Thermo Q Exactive HF operated with a data-dependent method as follows: MS full scans were performed in FTMS with resolution set to 120,000, from 300.0 to 1600.0 m/z, a maximum fill time of 50 ms, and an AGC target value of 3e6 ions. A maximum of 12 data-dependent MS2 scans was performed in the ion trap set to an AGC target of 1e5 ions with a maximal injection time of 100 ms. Precursor ion fragmentation was achieved with collision-induced fragmentation with a normalised collision energy of 27 and isolation width of 1.2 m/z. Charge states of 1 and ≥7 were rejected.

Data pre-processing: LC-MS metabolite data.

The LC-MS data were processed using Expressionist Refiner MS 11.0 (Genedata AG, Basel, Switzerland) and the settings described previously [8], with minor changes. In brief, the following activities and settings were modified: chromatogram alignment (RT search interval 0.5 min), peak detection (minimum peak size 0.03 min, gap/peak ratio 50%, smoothing window 5 points, centre computation by intensity-weighted method with threshold at 70%, boundary determination using inflection points), isotope clustering (RT tolerance at 0.015 min, m/z tolerance 5 ppm, allowed charges 1–4), filtering for a single peak not assigned to an isotope cluster, adduct detection and clusters grouping (RT tolerance 0.05 min, m/z tolerance 5 ppm, maximum intensity of side adduct 100,000%). Detailed instructions for using the software can be found in prior work [9].

Processing of 192 fractionated and four blank samples using Expressionist Refiner MS 11.0 resulted in the detection of 40,139 and 8,671 metabolite clusters in the positive and negative modes, respectively (Supplementary Data S14 and S15). Subsequently, the data were filtered as follows: to be considered protein bound, the maximum intensity of the metabolic mass feature present in the protein-containing fraction (fractions from A6 to C13) was required to be 10 times above the average blank intensity, 10 times above the maximum intensity of the non-protein control, and greater than 5000. We used an in-house algorithm for alternative adduct detection, allowing a 0.015 min RT deviation and a 0.005 Da mass

deviation and restricting a list of potential adducts to [M+H], [M+Na], [M+NH4] and [M-H], [M+HCOO] in the positive and negative modes.

Data pre-processing: LC-MS metabolite annotation.

All metabolite clusters were matched to in-house libraries of authentic reference compounds, allowing a 0.005 Da mass and dynamic retention time deviation (maximum 0.2 min). This enabled the identification of 74 metabolites present in the protein-containing fraction. Low abundance adducts and mass features detected as single intensity peaks were excluded from the list. The median intensity of three replicates was used to calculate the elution profile of 74 metabolites.

Data pre-processing: LC-MS/MS protein data.

Raw data were analysed using MaxQuant version 1.6.0.16 [3] and its built-in search engine, Andromeda [4]. The *S. cerevisiae* protein database, modified 19 February 2017 and containing 6049 identifications, was downloaded from Uniprot (http://www.uniprot.org/proteomes/UP000002311). The search also included a contaminant database. The MaxQuant internal algorithm was used to merge three independent replicates. Detailed MaxQuant settings and parameters are shown in Supplementary Data S16.

A total of 4009 protein groups were initially identified in 38 protein-containing fractions. The number of proteins decreased to 3982 when contaminants and decoy hits were filtered out (Supplementary Data S3). At least two unique peptides (or at least one unique and one razor peptide if they contributed to more than 25% unique sequence coverage) were required per protein group. Raw intensities acquired for each protein group were used for subsequent analysis.

Data pre-processing: protein and metabolite peak deconvolution.

Metabolite peaks not spanning at least two consecutive fractions were removed in separate replicates. The median profiles were then calculated with zero values replaced by NA values. The potential peaks were considered to be true if they fulfilled the following criteria: (i) the intensity of a potential peak was equal to or above 15% and 30% of the maximum peak intensity for metabolites and proteins, respectively; (ii) the minimum drop and rise in intensity between the peaks was higher than 30% of the previous peak's intensity; and (iii) the peak profile was recorded until it reached 20% and 25% of the maximum peak

intensity for metabolites and proteins, respectively. Protein and metabolite peaks that appeared in fewer than two consecutive fractions were excluded. Peak deconvolution resulted in a final list of 125 annotated metabolic feature peaks and 5834 protein peaks.

Data pre-processing: correlation of profiles.

The similarity between the deconvoluted elution profiles of annotated metabolites and proteins was determined by calculating the PCC (Supplementary Figure S23) (Supplementary Data S17). The integrated data set was further used to predict PM complexes.

Data pre-processing: untargeted analysis of mass features.

To determine a list of small molecules present in the protein-containing fractions, 40139 and 8671 molecules detected in the positive and negative modes, respectively, were filtered as described above. Subsequently, all mass feature peaks not spanning at least two consecutive fractions were removed in separate replicates. The PCC was calculated between the elution profiles of adducts associated with the same metabolite. Less abundant adducts were removed from the data set if they were correlated with highly abundant adducts of the same compound (PCC > 0.9). Subsequently, non-annotated, putative ligands were determined by restricting the list of detected compounds to those with reproducible elution profiles in at least two replicates (PCC > 0.9) (Supplementary Figure S24). In total, 821 and 195 putative ligands were detected in the positive and negative modes, respectively; after deconvolution, they created 1445 separate peaks distributed across all protein-containing fractions.

Data processing: determination of a list of proteins in a complex.

To determine a list of proteins in a complex, the apparent mass of a protein was calculated based on the elution profile of single peaks. A ratio of the apparent mass of a protein to the theoretical monomeric mass of a protein (referred to as the oligomeric state ratio) was then calculated [10] (Supplementary Data S8).

Data processing: determination of a list of known and putative protein–metabolite complexes.

The STITCH database was first restricted to proteins and metabolites quantified in the PROMIS experiment. Experimentally confirmed interactions passing the 800 confidence threshold were considered to be known PMIs. Confidence score is calculated based on a

cumulative data coming from multiple source e.g. experimental approaches, databases or data mining. 800 confidence score indicates that evidences coming from available sources are sufficient to conclude the interaction between molecules with probability of 80%. A subset of reported complexes contained 87 PMIs (Supplementary Data S9 and S10). Protein–metabolite interactions with experimental scores equal to or above 150 were considered to be putative. The combined score threshold was set to 400. Interactions with experimental scores above 800 were excluded. The final list contained 1122 putative PM complexes.

To determine the influence of using the Pearson correlation coefficient threshold on the false discovery rate, we (1) retrieved 87 reported true PMIs from the STITCH database, including only proteins and metabolites identified in our data. (2) We calculated Pearson correlation coefficient between metabolite and protein peaks, which were components of 87 reported true PMIs (Supplementary Data S9). (3) We calculated Pearson correlation coefficient between all metabolite and all protein peaks present in our data set (Supplementary Data S17). (4) We used Supplementary Data S17 to randomly pick 87 Pearson correlation coefficient values (100 times) for any given protein-metabolite pair. Next, in each iteration, randomly picked Pearson correlation coefficient values have been sorted in descending order. For each of the 87 protein-metabolite pairs, we calculated the median Pearson correlation coefficient of all of the iterations performed. (5) Finally, we compared Pearson correlation coefficients obtained for 87 true interactions retrieved from STITCH database (obtained in point 2) with median Pearson correlation coefficient of all of the iterations performed (obtained in point 4). Obtained values were used to determine true positive and false positive rates and to construct receiver operating characteristic (ROC) curve.

Moreover, we provide additional information in Supplementary Data S10 regarding the number of random pairs with a PCC above given threshold per permutation over 100 iterations. Based on this number, we calculated alternative False Positive Rate and False Disovery Rate for any given PCC threshold in a range from 0 to 1.

Overexpression and purification of Pnp1 and Pgk1.

Pnp1 and Pgk1 overexpressing yeast strains were purchased from Dharmacon and are part of the yeast ORF collection [2]. Yeast glycerol stock was streaked onto SD-Ura plates (SD/-Ura Broth, Takara, 630314, 2% Agar) and cultivated for 2 days at 28 °C. A single

colony was used for the inoculation of 25 mL of SD-Ura. The culture was incubated for 24 h at 28 °C with shaking. The next day, the culture was diluted to OD600 = 0.014 in SR-Ura (yeast synthetic drop-out medium without Uracil, Sigma, Y1501, yeast nitrogen base without amino acids, Sigma, Y0626, Raffinose, Sigma, R0250) and cultivated approx. 16 h to OD600 = 0.5–0.9. Protein expression was induced by the addition of a 1/3 volume of 3 × YPG (yeast extract, Sigma, Y1625, bacto peptone, Sigma, 91249, galactose, Sigma, G0750) pre-warmed to 28 °C, and cultivation was continued for 6 h. Cells were collected by centrifugation at 1500 g, 5 min, 4 °C. Cells were subsequently washed with ice-cold MilliQ water and centrifuged 5 min, 1500 g, 4 °C. The resulting yeast pellet was flash frozen in liquid nitrogen and stored at -80 °C until needed.

The collected cells were solubilised in an ice-cold Pnp1 lysis buffer consisting of 50 mM Tris-HCl pH 7.5, 150 mM NaCl, 5 mM DTT, 100 mM PMSF, 1 EDTA-free Protease Inhibitor Cocktail (MERCK, 11873580001). The Pgk1 lysis buffer consisted of 50 mM Tris-HCl pH 7.0, 50 mM NaCl, 1 mM EDTA, and 1x cOmplete EDTA-free Protease Inhibitor Cocktail (MERCK, 11873580001). Frozen (-20 °C) silica/zirconia beads (Biospec, 11079105z) were added to the cell slurry, and the yeasts were homogenised by bead beating 10 × 30 s at 20 Hz using a Retsch Mixer Mill MM 400. In between bead beating, the lysates were cooled for 1 min in an ice-water bath.

Cell debris and zirconia-silica beads were separated by a 5 min centrifugation at 4000 g and 4 °C. The supernatant was further centrifuged for 10 min, 20000 g, at 4 °C to obtain the soluble protein extract. The protein extract was further incubated for 1 h at 4 °C with pre-washed (4 times with 50 mM Tris-HCl pH 7.5, 150 mM NaCl and one time with lysis buffer in case of Pnp1 and with 50 mM Tris-HCl pH 7.0, 150 mM NaCl, 1 mM EDTA in case of Pgk1) IgG Sepharose® 6 Fast Flow beads (GE Healthcare, 17-0969-01). Beads were transferred to a Mobicol “Classic” filter (35 µm pore size, MoBiTec, M1002S and M513515) and washed with 30 mL of wash buffer (Pnp1: 50 mM Tris-HCl pH 7.5, 150 mM NaCl; Pgk1: 50 mM Tris-HCl pH 7.0, 150 mM NaCl, 1 mM EDTA) and 5 mL of Protease 3C cleavage buffer (Pnp1: 50 mM Tris-HCl pH 8.0, 150 mM NaCl; Pgk1: 50 mM Tris-HCl pH 7.0,150 mM NaCl, 1 mM EDTA). The lower cap of the Mobicol “Classic” was closed, and 400 µL of Protease 3C cleavage buffer, containing 10 U of Protease 3C (ThermoFisher Scientific, 88947) was added.

Samples were incubated for 16 h at 4 °C on a rotary wheel. Eluates were collected by centrifuging 30 s, 200 g, at 4 °C. An additional 200 µL of Protease 3C cleavage buffer was added to the beads and centrifugation was repeated. In total, 600 µL of the eluate was collected. To remove the Protease 3C, 600 µl of the eluate was incubated with glutathione-agarose (Sigma, G4510) beads for 1 h at 4 °C on a rotary wheel. The solution was transferred to a Mobicol "Classic" filter (35 µm pore size) and centrifuged 30 s, 200 g, at 4 °C. The purity of the Pgk1 was determined using SDS-PAGE (Supplementary Figure S25).

Approximately 0.5 mL of recombinant Pnp1 was separated by SEC. Fractionation was performed on an HPLC, Sepax, Zenix SEC-300, column, 3 µm, 300 A 10/300 connected to an ÄKTA explorer 10 (GE Healthcare Life Science, Little Chalfont, UK) at 1 mL/min flow rate, 4 °C. Equilibration of the column and separation was performed using 50 mM Tris-HCl pH 8.0, 150 mM NaCl. Twelve fractions of 1 ml were collected from the 8 ml to 20 ml elution volume, frozen by snap freezing in liquid nitrogen, and stored at –80 °C. The purity of the protein was confirmed using SDS-PAGE (Supplementary Figure S26). Fractions A9 and A10 were combined, concentrated using 10 kDa MWCO Amicon Filter (Sigma, Z706345), and used for enzymatic assay. The procedure was repeated until a sufficient amount of protein was reached.

Pnp1 enzymatic assay.

The method for Pnp1 enzymatic activity measurement was adapted from previous studies [11]. Briefly, the concentration of hypoxanthine formed from inosine was quantified using LC-MS/MS [12] and processed using Expressionist Refiner MS 11.0 (Genedata AG, Basel, Switzerland), as described above for the PROMIS data set. The maximum peak intensity was used for relative quantification of chemical compounds (Supplementary Data S18). Inosine at 200 µM and 500 µM was used as a PNP1 substrate and 100 mM ammonium phosphate pH 7.5 as phosphate group donor. The activity of Pnp1 was measured in triplicate in the presence and absence of 100 µM xanthine. Each enzymatic reaction was initiated by adding 0.05 µg protein per well and running it for 5 min. A hypoxanthine calibration curve (3.15–0.1 µM hypoxanthine) was prepared in parallel to determine the amount of product formed in the enzymatic reaction. Inhibition was calculated in relation to Pnp1 activity in the absence of xanthine.

Preparation of native *S. cerevisiae* lysates for affinity purification.

The YSBN2 strain was cultivated at 28 °C with moderate shaking until it reached the logarithmic phase (OD600 = 0.3–0.5). The cells were collected by centrifugation (4000 g, 4 °C, 5 min), washed with ice-cold water, and centrifuged again (4000 g, 4 °C, 5 min). The pellets were snap frozen in liquid nitrogen and stored at -80 °C until needed. A 5 mL volume of ice-cold zirconia/silica beads (0.5 mm) and 4 mL of AP lysis buffer (50 mM Tris-Cl pH 7.5, 1.5 mM $MgCl_2$, 150 mM NaCl, 5 mM DTT, 1 mM PMSF, 1x cOmplete EDTA-free Protease Inhibitor Cocktail (MERCK, 11873580001), 0.1 mM Na_3VO_4, 1 mM NaF) was added per pellet of 600 mL yeast culture. The cells were homogenised by bead beating 10 × 30 s at 25 Hz using a Retsch Mixer Mill MM 400. In between bead beating, the lysates were cooled for 1 min in an ice-water bath. The cell debris and zirconia-silica beads were separated from the lysate by a 5 min centrifugation at 1500 g, 4 °C. The supernatant was ultracentrifuged for 1 h at 4 °C and 35000 rpm (max 148862 g, avg. 116140 g) to obtain a water-soluble molecule extract.

Affinity purification using Ser-Leu agarose beads.

Custom Ser-Leu agarose beads with molecules coupled via either the NH2 group of serine or the COOH group of leucine were purchased from Cube Biotech (Monheim, Germany). Prior to affinity purification, the beads were equilibrated by washing them 5 times with an AP lysis buffer (50 mM Tris-Cl pH 7.5, 1.5 mM $MgCl_2$, 150 mM NaCl, 5 mM DTT, 1 mM PMSF, 1x cOmplete EDTA-free Protease Inhibitor Cocktail (MERCK, 11873580001), 0.1 mM Na_3VO_4, 1 mM NaF). A 2 mL volume of water-soluble molecule extract (approx. 15 mg of protein) was combined with 300 µL of Ser-Leu agarose beads or “empty” agarose beads (negative control). The mixture was incubated for 1 h on a rotating wheel at 4 °C and then split into three Mobicol “Classic” columns with 35 µm pore size filters and washed with 10 mL of washing buffer (50 mM Tris-Cl pH 7.5, 1.5 mM $MgCl_2$, 500 mM NaCl). A 400 µL volume of 10 µM Ser-Leu dissolved in an elution buffer (50 mM Tris-Cl pH 7.5, 1.5 mM $MgCl_2$, 150 mM NaCl) was added to the beads, which were then incubated for a 30 min on a rotary wheel at 4 °C. The eluate was collected using a vacuum manifold system, and the procedure was repeated using 100 µM and 1 mM Ser-Leu. Finally, 400 µL of elution buffer was added to the beads, and the mixture was heated for 10 min at 99 °C using a benchtop Thermomixer Compact (ThermoFisher). The eluate was collected using a vacuum manifold system. Separate eluates were dried using a centrifugal evaporator and stored at -80 °C,

followed by MTBE-based biomolecule extraction. Proteins were extracted as described above. A total of 36 eluates were analysed using LC-MS/MS proteomics and processed as described above, with minor changes. The gradient was proportionally shortened to 30 min. Data were pre-processed using MaxQuant (Supplementary Data S19).

Affinity purification using Ser-Leu agarose beads: normalisation and target identification.

Initially, 1153 protein groups were identified in 36 eluates. The number of proteins decreased to 913 as the contaminants and decoy hits were filtered out (Supplementary Data S20). At least two unique peptides were required per protein group. The Log2 transformed LFQ intensities acquired for each protein group were used for subsequent analysis. Overall, 162 proteins were significantly enriched in the corresponding eluates from the agarose beads when compared to the empty beads (ANOVA $p < 0.05$, Tukey's $p < 0.05$) (Supplementary Data S21 and S22). A list comprising only proteins enriched in both N-Ser-Leu and Ser-Leu-C was constructed.

Thermal proteome profiling of the Ser-Leu-treated cell extracts.

Thermal proteome profiling of Ser-Leu-treated cell extracts was performed as described earlier [13]. Briefly, *S. cerevisiae* was cultured and harvested as described above. The pellet from a 600 mL yeast culture was resuspended in 5 mL of ice-cold zirconia/silica beads (0.5 mm) and 4 mL of an AP lysis buffer (50 mM HEPES pH 7.5, 1.5 mM $MgCl_2$, 150 mM NaCl, 5 mM DTT, 1 mM PMSF, 1x cOmplete EDTA-free Protease Inhibitor Cocktail (MERCK, 11873580001)). Cells were homogenised by bead beating 10 × 30 s at 25 Hz using a Retsch Mixer Mill MM 400. In between bead beating, the lysates were cooled for one min in an ice-water bath.

The cell debris and zirconia-silica beads were separated by a 5 min centrifugation at 1500 g, 4 °C. The supernatant was further centrifuged for 20 min at 20000 g at 4 °C to obtain a soluble protein extract. The extract was subsequently loaded onto an Amicon Ultra-15 Centrifugal Filter Unit (10 kDa MWCO) and centrifuged for 15 min at 4 °C, 4000 g. The protein concentration of the supernatant was determined using the Bradford assay. Ser-Leu or H_2O alone as a vehicle was added to the cell extract containing 5 mg of protein. The extract was then incubated for 20 min at 30 °C, divided into 10 aliquots of 100 µl, and transferred into 0.2 mL PCR tubes. Samples containing Ser-Leu and the vehicle were heated in parallel

for 3 min to the desired temperature and then incubated for 3 min at room temperature. The samples were then centrifuged 20 min, 20000 g, at 4 °C. The supernatant was transferred to fresh Eppendorf tubes.

The soluble proteins were precipitated in 80% MS grade acetone at -20 °C for 16 h, followed by centrifugation for 20 min, 20000 g, at 4 °C. The acetone solution was discarded, and the protein pellet was dried using a centrifugal evaporator. Samples were prepared for MS analysis and analysed as described above using a 120 min gradient. The experiment was performed in triplicate. Each replicate was independently treated with molecules and subsequently heated to the desired temperature. Data were pre-processed using MaxQuant (Supplementary Data S23).

Thermal proteome profiling of Ser-Leu treated cell extracts: normalisation and target identification.

Initially, 2073 protein groups were identified in 60 samples. The number of proteins decreased to 1798 as contaminants and decoy hits were filtered out (Supplementary Data S24). At least two unique peptides were required per protein group. Raw intensities were median normalised per temperature. The protein abundance was calculated relative to intensity at the lowest temperature. Significant changes in the melting curves were determined using a TPP package available on Bioconductor and NPARC [14] (Supplementary Data S25).

Microscale thermophoresis.

Microscale thermophoresis measurements were performed using a Monolith NT.115 instrument (Nanotemper). In all experiments, affinity binding was determined using standard capillaries, 100–150 nM labelled protein, medium MST laser power, and LED power optimised to yield emission intensities around 200 AU. Commercially available Pgk1 (Sigma P7634) was labelled in a Tris buffer (50 mM Tris-HCl pH 7.0, 50 mM NaCl, 10 mM $MgCl_2$) using a RED-MALEIMIDE labelling kit (Cysteine reactive, MO-L004) according to the manufacturer's manual. Prior to labelling, cysteine residue of Pgk1 was reduced using 5 mM TCEP to assure labelling efficiency.

Ligands Ser-Leu, Tyr-Asp, ATP, and serine were dissolved in the Tris buffer (50 mM Tris-HCl pH 7.0, 50 mM NaCl, 10 mM $MgCl_2$). To diminish the amount of protein sticking

to capillaries, binding mixture was supplemented with 0.01% Tween 20. Binding was tested in three distinct replicates. At least 12 points from each titration were used to determine the binding (Supplementary Data S26-27). MO Affinity Analysis software was used to calculate the K_d of the binding. Results were subsequently exported and visualised using Adobe Illustrator CS5.

Pgk1 enzymatic assay.

Pgk1 activity was assayed using an optimised stopped assay, and the product was determined by an enzyme-cycling system, as described earlier, with minor modifications [15,16]. Aliquots of the recombinant protein were appropriately diluted and, together with G3P standards ranging from 0 to 500 µM, were incubated in 100 mM Tricine/KOH, pH 8.0, 20 mM $MgCl_2$, 50 mM KCl, 2 mM EDTA, 0.05% Triton X100, 4 mM 3PGA, 5 mM dithiothreitol, 6 mM NADH, 1 unit mL^{-1} NAD-dependent glyceraldehyde-3-P dehydrogenase, 1 unit mL^{-1} triose-P isomerase, 2 unit mL^{-1} glycerol-3-P dehydrogenase, and varying concentrations of ATP: 0 (blank), 1 mM, 5 mM, or 50 mM (maximal activity), in a final reaction volume of 20 µL. The effect of the presence of Ser-Leu in the Pgk1 activity was tested by the addition of 0 to 1 mM Ser –Leu in the reaction mix.

After 20 min at 25 °C, the reaction was stopped by the addition of 20 µL of 0.5 M HCl and 0.1 M Tricine/KOH pH 9.0 and incubated at room temperature for 10 min. The stopped assay was neutralised, and the G3P produced was measured by the glycerol-3-P dehydrogenase/glycerol-3-P oxidase enzyme-cycling reaction, as described earlier [16]. To do this, we added 20 µL of 0.5 M NaOH and 50 µL of the determination mix, containing 200 mM Tricine/KOH, pH 8.0, 4 mM $MgCl_2$, 1.2 mM NADH, 2 unit mL^{-1} glycerol-3-P dehydrogenase, and 5 unit mL^{-1} glycerol-3-P oxidase. The decrease in the NADH concentration was followed at 340 nm and 30 °C until the rates were stabilised (Supplementary Data S28).

Estimation of Ser-Leu level in yeast

Concentration of Ser-Leu in *S. cerevisiae* was estimated by spiking different amounts of non-labeled Ser-Leu (from 100 nM to 10 mM) into metabolic extract of 13C labelled S288c yeast culture (Supplementary Data S29). 6.75 mL of yeast culture (OD_{600} = 3.0) was collected by centrifugation (4000 g, 5 min) and subjected to MTBE extraction (see above). Metabolic extract was suspended in 400 µl of LC-MS grade water, supplemented with

different amounts of non-labeled Ser-Leu and analysed using LC-MS (see above). To extract peaks, obtained chromatograms were processed using Expressionist Refiner MS 11.0 (Genedata AG, Basel, Switzerland) and the settings described previously (see above). Peaks intensities corresponding to spiked Ser-Leu (m/z = 219.1336) were used for preparation of calibration curve. Obtained calibration curve had a linear trend from 100 nM to 10 µM with R^2 = 0.9999, slope (a) equal to 74711973.24 and interception (b) equal to -6342084.833. Average intensity of 13C-labelled Ser-Leu was $1.13x10^7$ (n = 4) and this value was used for further calculations. To calculate concentration of Ser-Leu per cell volume we used previously published estimations of yeast cell volume ($4.2x10^{-14}$ L/cell) [17] and number of cells per OD unit ($2x10^7$ cells/mL) [18].

Dipeptide and amino acid accumulation during growth.

The YSBN2 strain of *S. cerevisiae* was cultivated at 30 °C using SC medium (0.5% glucose), with moderate shaking until it reached the stationary phase. Glucose was re-introduced by adding 80 mL of fresh media at 30 °C to 50 mL of cell cultures maintained at 30 °C [19]. Cultures were immediately transferred to 6-well plates and placed into temperature-controlled shakers. Equal aliquots from each culture were collected at specific time points (immediately after mixing yeast culture with pre-treated fresh media, and later after 15 min to 1440 min of cultivation) by quenching metabolites in dry-ice-level cold methanol, followed by centrifugation (4,000 g 4 °C, 5 min). Supernatant was disposed and yeast pellet was snap-frozen using liquid nitrogen. Polar metabolites were extracted and further analysed using LC-MS as described above. Peaks extraction from obtained chromatograms was performed using Expressionist Refiner MS 11.0 (Genedata AG, Basel, Switzerland) and the settings described above. Mass features were annotated using reference compound library (limited to dipeptides and amino acids) as described above. Data filtering and normalisation was performed as follows. All mass features, which were not detected (intensity threshold ≥ 10,000) in at least 33% samples were excluded from the further analysis. Intensities of remaining mass features were median normalized (Supplementary Data S30). For the purpose of data presentation, normalized intensities were transformed as follows. First, a mean of 3 biological replicates was calculated. Next, changes in metabolites intensities were calculated relative to time point 0. Finally, ratios were log transformed (log base 2).

Yeast growth upon Ser-Leu supplementation.

The YSBN2 strain of *S. cerevisiae* was cultivated at 30 °C using SC medium (0.5% glucose), with moderate shaking until it reached the stationary phase. Culture was diluted to OD_{600} nm = 0.05 in fresh SC media (0.5% glucose) and treated with either mock, 1 mM Ser-Leu or a mixture of 1 mM serine and 1 mM leucine. Growth of three independent replicas was followed for 75h by monitoring changes at OD_{600} nm using Biotek Synergy H1 microplate reader (Supplementary Data S31).

Changes in metabolism upon Ser-Leu supplementation (^{13}C-isotope-labeling experiment) followed by GC-MS analysis.

The YSBN2 strain of *S. cerevisiae* was cultivated at 30 °C using SC medium (0.5% glucose), with moderate shaking until it reached the stationary phase (referred as pre-culture). Chemical treatment was applied by adding 68 mL of pre-warmed to 30 °C fresh media (0.5% 13C Glucose) supplemented with either mock, 100 µM Ser-Leu or a mixture of 100 µM serine and 100 µM leucine to 136 mL of yeast pre-culture. Three independent replicas were prepared for each time point and chemical treatment. Forty-five cultivation flasks, containing 12 mL of chemically treated yeast cultures were placed into temperature-controlled shakers (30 °C, 140 RPM). Equal aliquots from each culture were collected at specific time points (5 min to 80 min after chemical treatment) by quenching metabolites in dry-ice-level cold methanol, followed by centrifugation (4,000 g 4 °C, 5 min). Supernatant was disposed and yeast pellet was snap-frozen using liquid nitrogen. Primary metabolites were extracted as follows. Frozen yeast pellet was solubilized in 1 mL of ice-cold MeOH (LC-MS grade) using vortex mixer. It was followed by cell grinding using Zirconia/Silica (0.5 mm diameter), 10 min sonication and 10 min incubation on ice. Finally, 0.25 mL of water was added followed by centrifugation (20,800 g, 4 °C, 5 min). Equal volume of supernatant was collected from each sample, dried in a centrifugal evaporator and stored at –80 °C until the samples were processed further.

Gas chromatography-mass spectrometry (GC-MS) profiling of metabolite extracts involved a two-step chemical derivatization described previously in detail [20,21]. Briefly, dried metabolic extracts were first methoxyaminated by agitating for 90 min at 30 °C with 40 µl of fresh metoxyamination reagent (40 mg mL^{-1} methoxamine hydrochloride in pure pyridine). Next, metabolites were trimethylsilylated by agitating 30 min at 37 °C with 80 µl of N-methyl-N-trymethylsilyl)-trifluoroacetamide-mix containing retention-index standards.

Splitless injection of 1 µl and data acquisition was performed using Pegasus III time-of-flight mass spectrometer (LECO Instrumente GmbH, Mönchengladbach, Germany; http://www.leco.de) coupled to Agilent 6890N24 gas chromatograph (Agilent Technologies, Böblingen, Germany; http://www.agilent.com) as described previously [20,22].

GC-MS chromatograms were visually controlled, baseline corrected and exported in NetCDF file format using ChromaTOF software (LECO, St. Joseph, USA) and further processed using TagFinder, which generated a peak intensity matrix containing all mass isotopomers of mass fragments [23,24]. Next, manually supervised annotation of metabolites was performed. For this purpose, retention indices (RI) and mass-spectra from the Golm Metabolome Databse (GMD, http://gmd.mpimp-golm.mpg.de/) were used [25,26]. Retention indices were calculated within each sample using alkanes provided by the retention index standard mixture during derivatization. Data matrix was manually supervised curated for targeted analytes extracting isotopomer-traces of known molecular structure and therefore atomic composition [27]. Resulted matrix was processed using CORRECTOR software tool (https://www.mpimp-golm.mpg.de/10871/Supplementary_Materials), which calculated the sum of mass isotopomer intensities and the ^{13}C enrichments of mass fragments [28-30].

Analysed were changes in relative metabolite level (here described as total intensity) and redistribution of carbon isotope (enrichment level [%] multiplied by relative metabolite level, here described as 13C fraction intensity) (Supplementary Data S32). Note that depicted are relative, not the absolute, levels of metabolites.

Changes in metabolism upon Ser-Leu supplementation (^{13}C-isotope-labeling experiment) followed by LC-MS analysis.

The YSBN2 strain of *S. cerevisiae* was cultivated at 30 °C using SC medium (0.5% glucose), with moderate shaking until it reached the stationary phase (referred as pre-culture). Chemical treatment was applied by adding 20 mL of pre-warmed to 30 °C fresh media (0.5% 13C Glucose) supplemented with either mock, 100 µM Ser-Leu or a mixture of 100 µM serine and 100 µM leucine to 20 mL of yeast pre-culture. Cultures were immediately transferred to 6-well plates and placed into temperature-controlled shakers. Three independent replicas were prepared for each time point and chemical treatment. Equal aliquots from each culture were collected at specific time points (5 min to 240 min after chemical treatment) by centrifugation (4,000 g 4 °C, 5 min). Supernatant was disposed and

yeast pellet was snap-frozen using liquid nitrogen. Polar metabolites were extracted and further analysed using LC-MS as described above. Peaks extraction from obtained chromatograms was performed using Expressionist Refiner MS 11.0 (Genedata AG, Basel, Switzerland) and the settings described above. Mass features were annotated using reference compound library as described above. All mass features, which were not detected (intensity threshold ≥ 10,000) in at least 20% samples were excluded from the further analysis. Matrix containing annotated mass features was processed using CORRECTOR software tool (https://www.mpimp-golm.mpg.de/10871/Supplementary_Materials), which calculated the sum of mass isotopomer intensities and the ^{13}C enrichments of compounds.

Analysed were changes in relative metabolite level (here described as total intensity) and redistribution of carbon isotope (enrichment level [%] multiplied by relative metabolite level, here described as 13C fraction intensity) (Supplementary Data S33). Note that depicted are relative, not the absolute, levels of metabolites.

Supplementary Figures

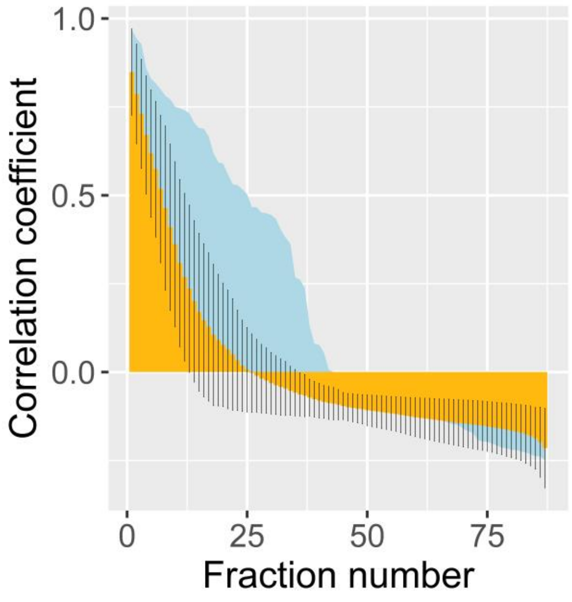

Supplementary Figure S1. Distribution of the Pearson correlation values measured for the 87 known protein – metabolite interactions (dark blue) vs. 87 random correlation values (yellow bars, average n = 100). Error bars represent standard deviation.

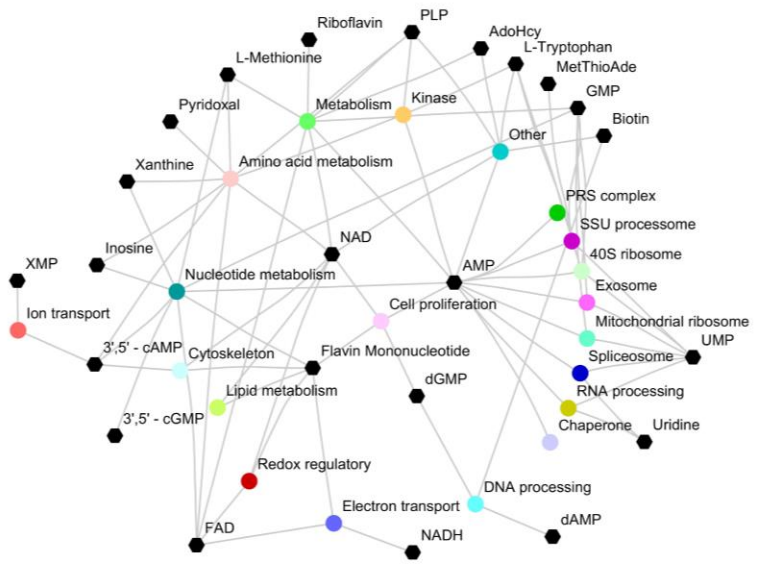

Supplementary Figure S2. Interaction network of 225 STITCH predicted protein – metabolite interactions validated in this study. Edges represent PMIs and were imported from STITCH based on the experimental evidence (score ≥ 400). Proteins were grouped based on their properties. Metabolite abbreviations: AdoHcy – adenosyl homocysteine, AMP – adenosine monophosphate, MetThioAde – methylthioadenosine, PLP – pyridoxal phosphate, UMP – uridine monophosphate, XMP – xanthine monophosphate.

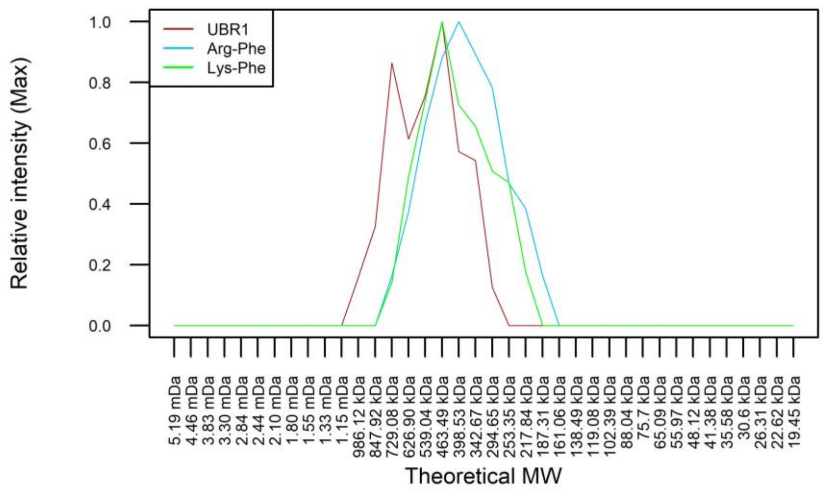

Supplementary Figure S3. Elution profiles of UBR1, Arg-Phe and Lys-Phe. Intensity was calculated relative to the maximum intensity of the molecule in the protein containing fractions. Theoretical molecular weight (MW) was estimated using reference proteins.

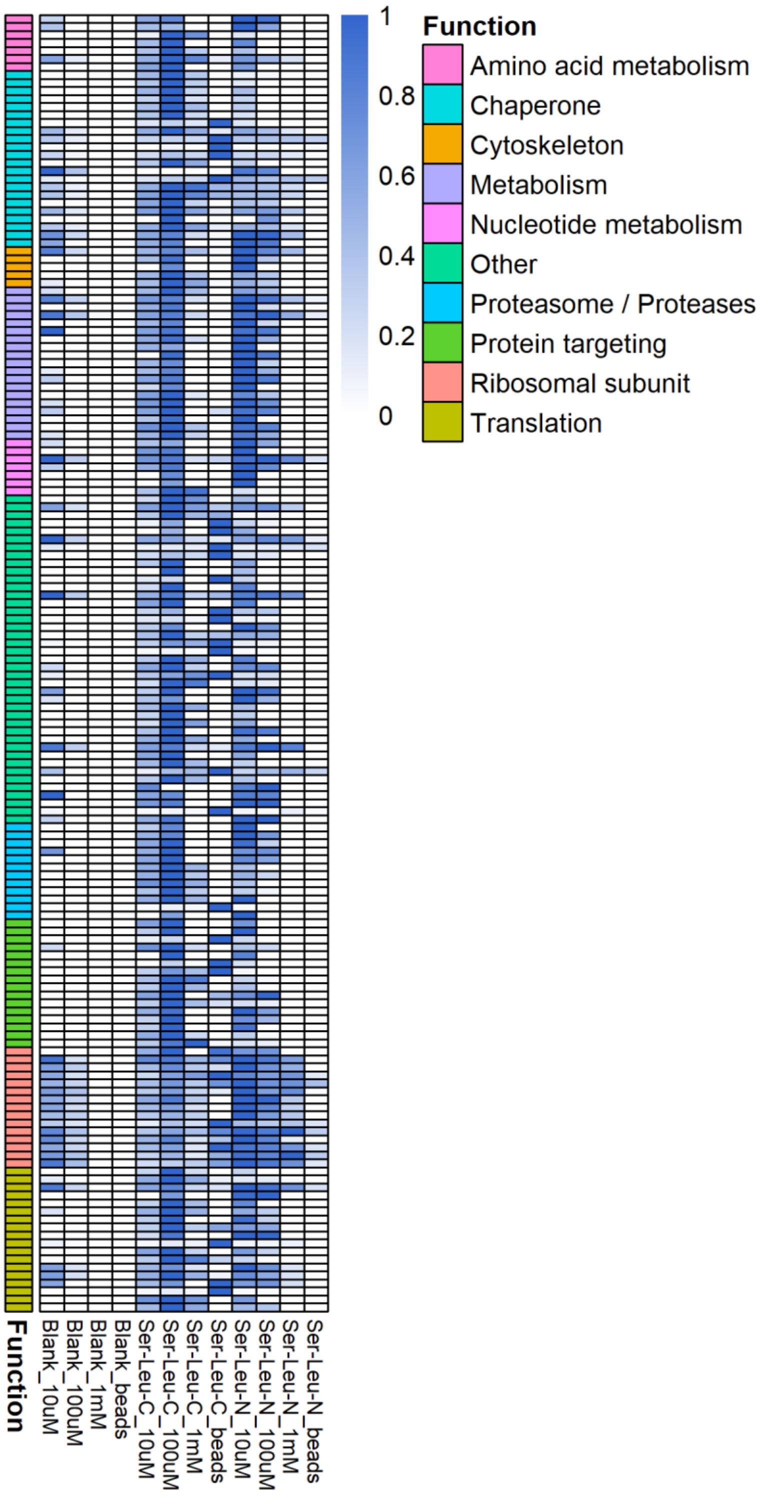

Supplementary Figure S4. Heatmap showing relative abundance (maximum of mean intensity) of 162 proteins significantly enriched in AP with Ser-Leu coupled agarose beads. Proteins were grouped by properties and marked using different colors. Blank samples correspond to empty beads controls. 4 consecutive washes with increasing concentration of Ser-Leu were performed to delineate targets of Ser-Leu.

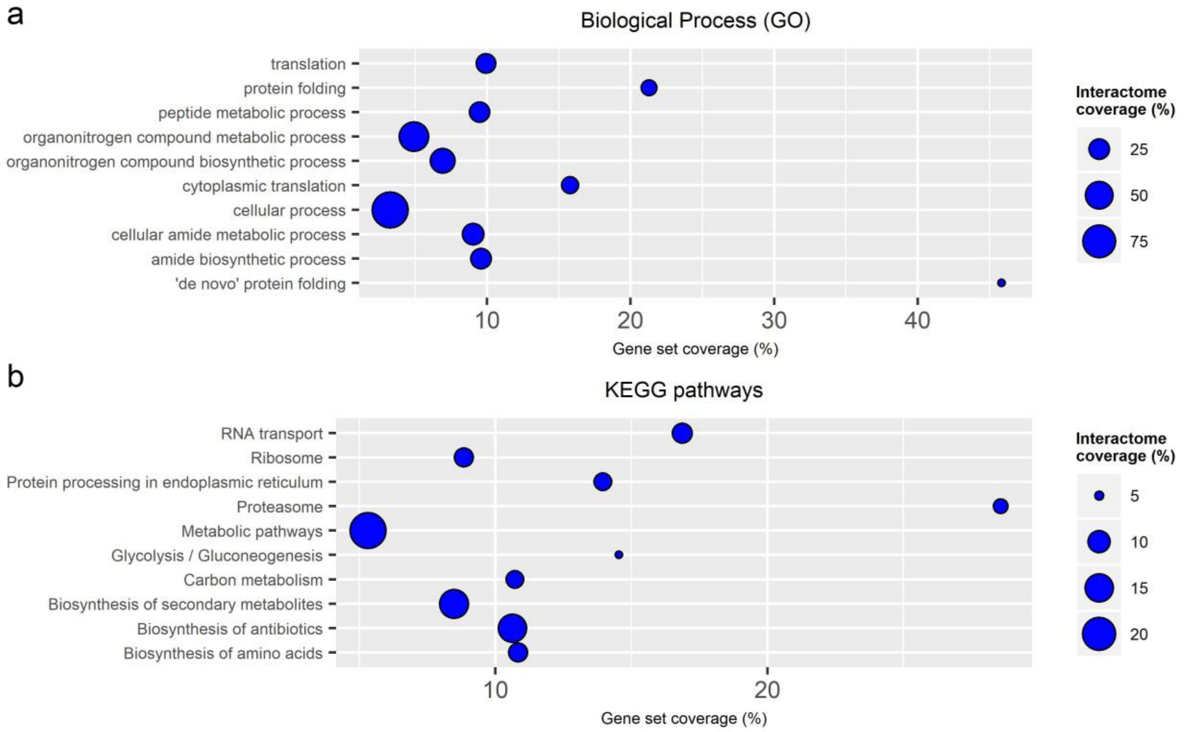

Supplementary Figure S5. Enrichment analysis of proteins significantly enriched in AP with Ser-Leu coupled agarose beads. a GO enrichment analysis. **b** KEGG pathways enrichment analysis. Top 10 most enriched processes are depicted (FDR < 0.05). Interactome coverage indicates relative coverage of the queried proteins. Gene set coverage indicates relative coverage of the respective enrichment category by queried proteins.

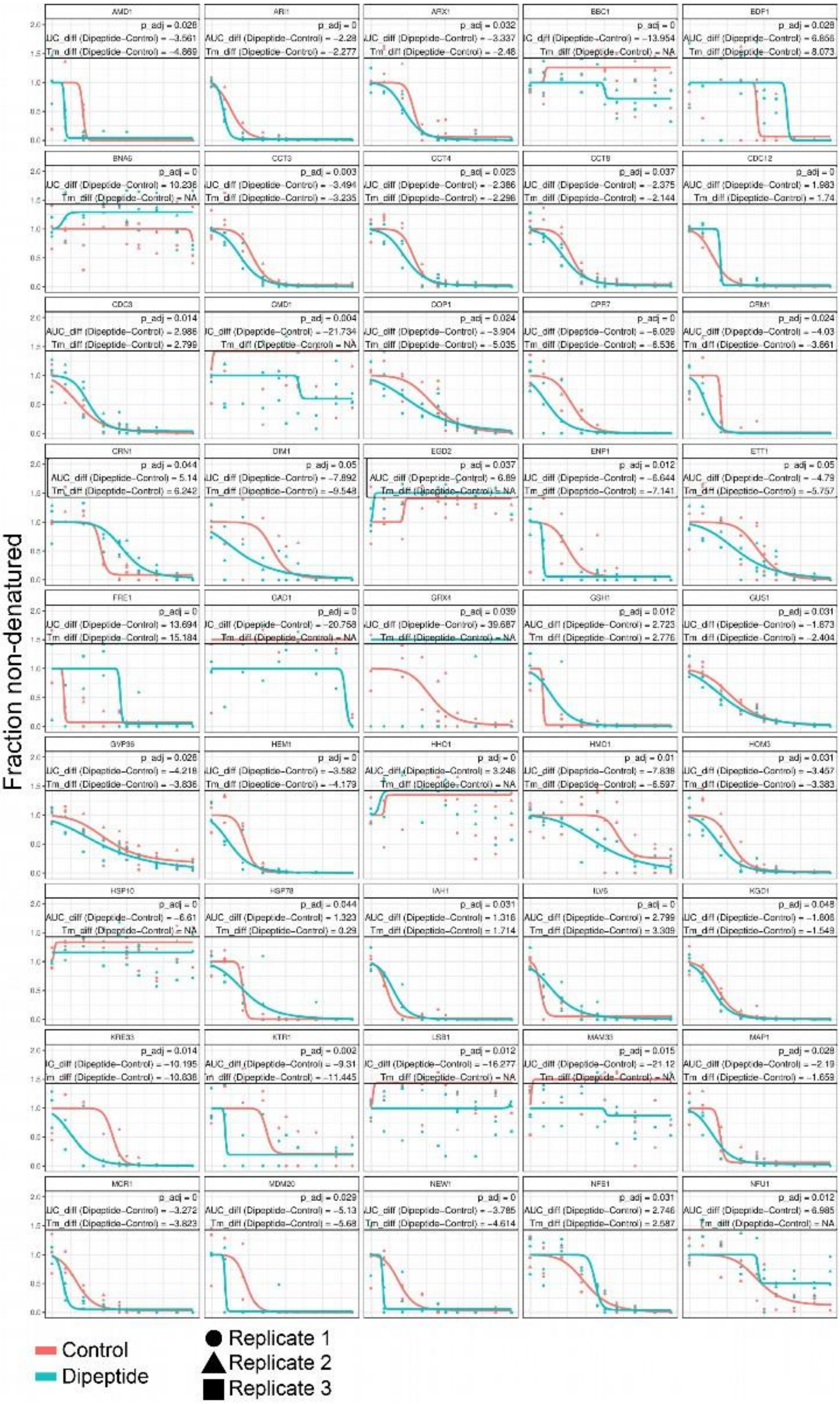
Fraction non-denatured
Control
Dipeptide
Replicate 1
Replicate 2
Replicate 3

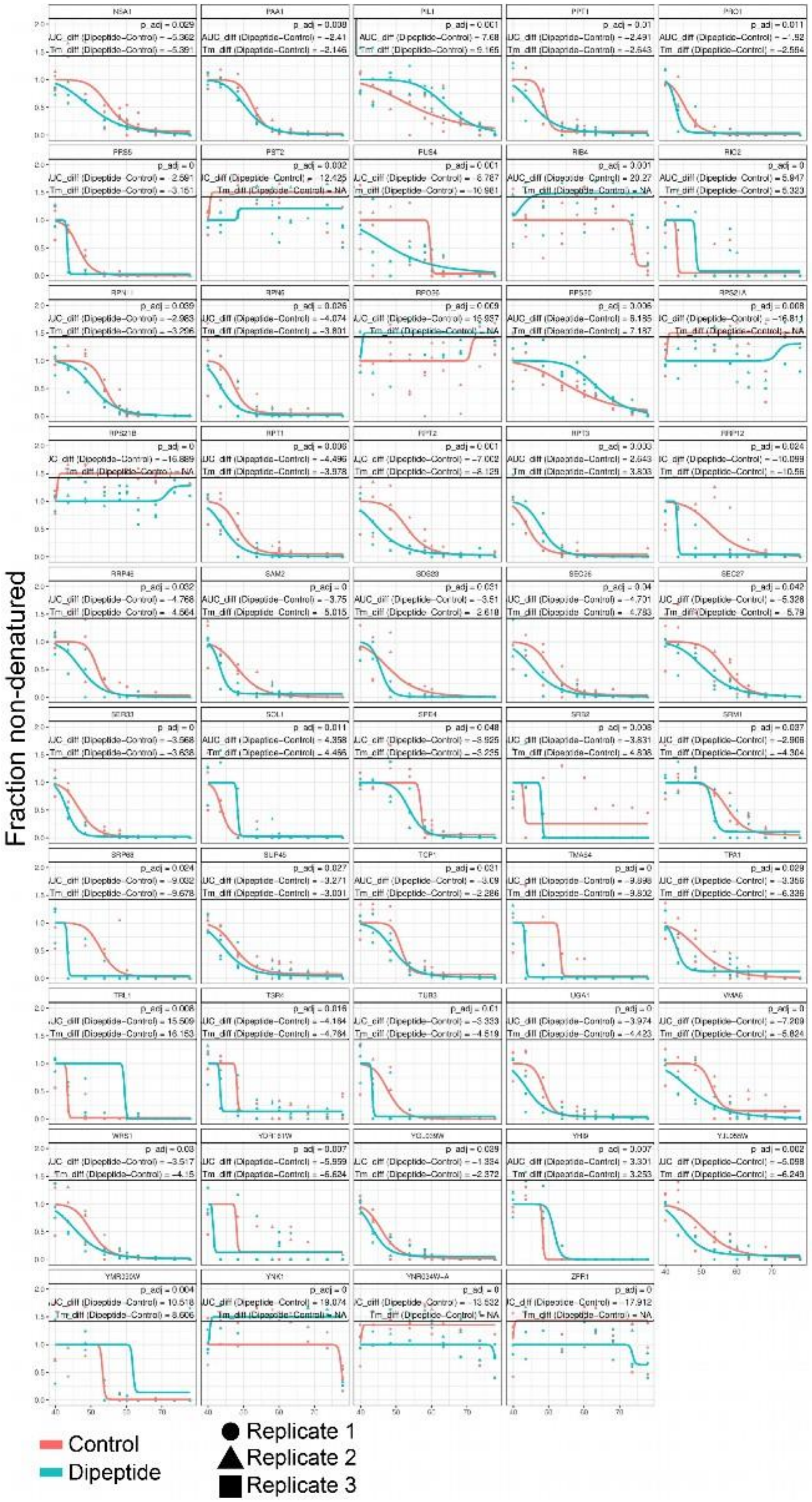
Fraction non-denatured
Control
Dipeptide
Replicate 1
Replicate 2
Replicate 3

Supplementary Figure S6. Melting profiles of putative Ser-Leu targets. NPARC method was used to determine significant changes in the melting profiles upon treatment with Ser-Leu. Turquois and red were used to mark melting profiles of treated and control samples, respectively.

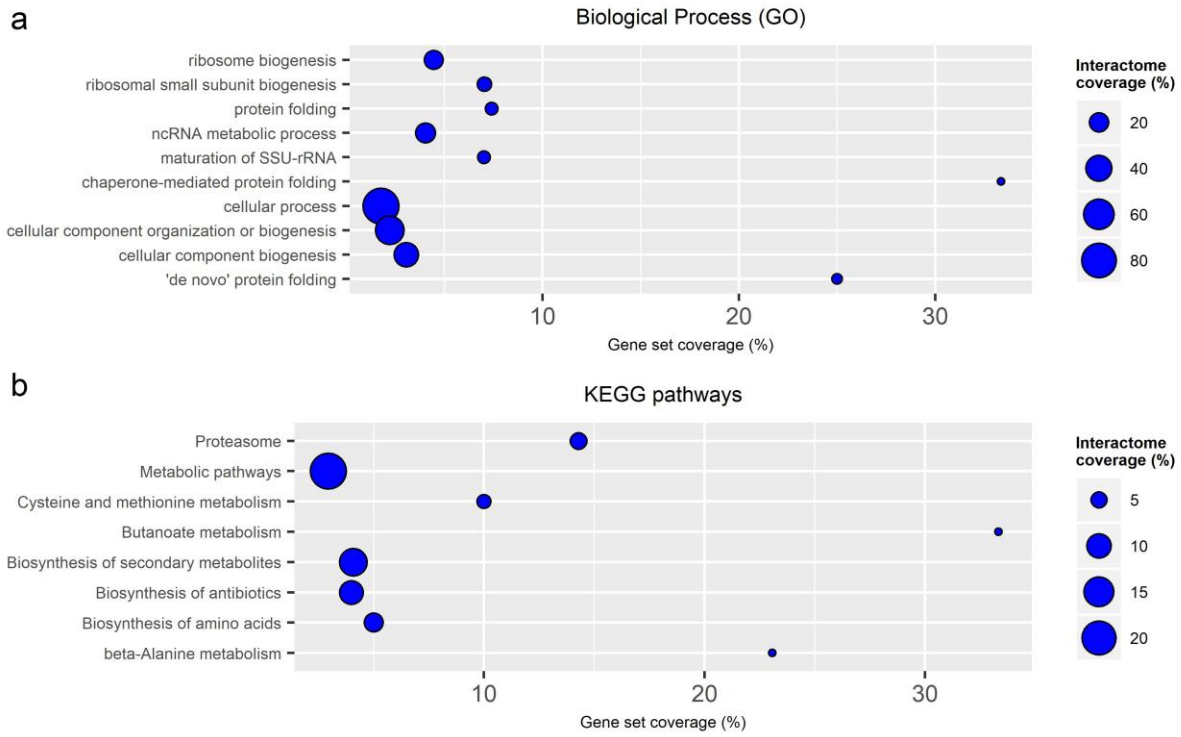

Supplementary Figure S7. Enrichment analysis of proteins, which melting curve was significantly affected by addition of Ser-Leu. a GO enrichment analysis. **b** KEGG pathways enrichment analysis. Top 10 most enriched processes are depicted (FDR < 0.05). Interactome coverage indicates relative coverage of the queried proteins. Gene set coverage indicates relative coverage of the respective enrichment category by queried proteins.

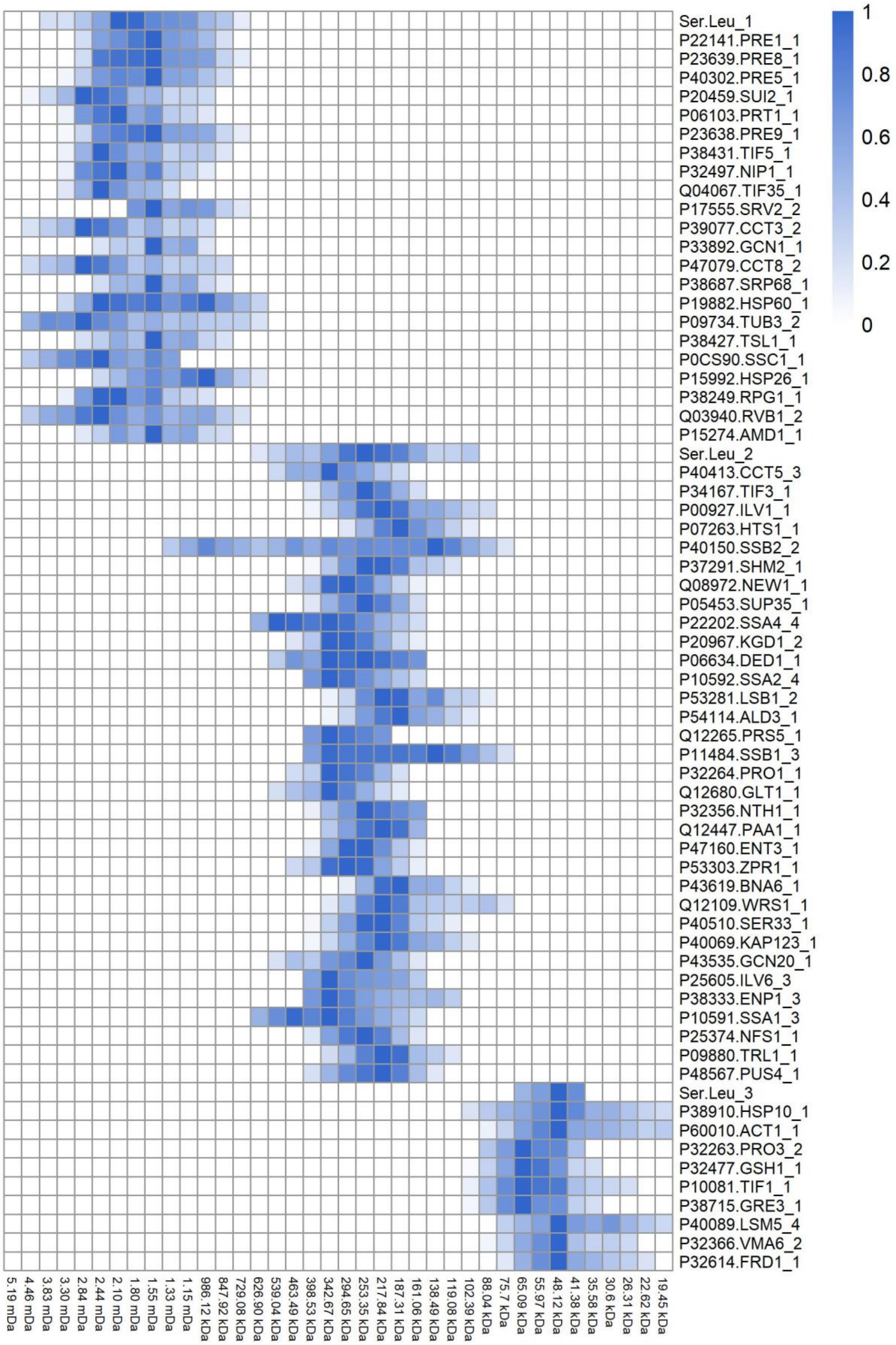

Ser.Leu_1
P22141.PRE1_1
P23639.PRE8_1
P40302.PRE5_1
P20459.SUI2_1
P06103.PRT1_1
P23638.PRE9_1
P38431.TIF5_1
P32497.NIP1_1
Q04067.TIF35_1
P17555.SRV2_2
P39077.CCT3_2
P33892.GCN1_1
P47079.CCT8_2
P38687.SRP68_1
P19882.HSP60_1
P09734.TUB3_2
P38427.TSL1_1
P0CS90.SSC1_1
P15992.HSP26_1
P38249.RPG1_1
Q03940.RVB1_2
P15274.AMD1_1
Ser.Leu_2
P40413.CCT5_3
P34167.TIF3_1
P00927.ILV1_1
P07263.HTS1_1
P40150.SSB2_2
P37291.SHM2_1
Q08972.NEW1_1
P05453.SUP35_1
P22202.SSA4_4
P20967.KGD1_2
P06634.DED1_1
P10592.SSA2_4
P53281.LSB1_2
P54114.ALD3_1
Q12265.PRS5_1
P11484.SSB1_3
P32264.PRO1_1
Q12680.GLT1_1
P32356.NTH1_1
Q12447.PAA1_1
P47160.ENT3_1
P53303.ZPR1_1
P43619.BNA6_1
Q12109.WRS1_1
P40510.SER33_1
P40069.KAP123_1
P43535.GCN20_1
P25605.ILV6_3
P38333.ENP1_3
P10591.SSA1_3
P25374.NFS1_1
P09880.TRL1_1
P48567.PUS4_1
Ser.Leu_3
P38910.HSP10_1
P60010.ACT1_1
P32263.PRO3_2
P32477.GSH1_1
P10081.TIF1_1
P38715.GRE3_1
P40089.LSM5_4
P32366.VMA6_2
P32614.FRD1_1
5.19 mDa
4.46 mDa
3.83 mDa
3.30 mDa
2.84 mDa
2.44 mDa
2.10 mDa
1.80 mDa
1.55 mDa
1.33 mDa
1.15 mDa
986.12 kDa
847.92 kDa
729.08 kDa
626.90 kDa
539.04 kDa
463.49 kDa
398.53 kDa
342.67 kDa
294.65 kDa
253.35 kDa
217.84 kDa
187.31 kDa
161.06 kDa
138.49 kDa
119.08 kDa
102.39 kDa
88.04 kDa
75.7 kDa
65.09 kDa
55.97 kDa
48.12 kDa
41.38 kDa
35.58 kDa
30.6 kDa
26.31 kDa
22.62 kDa
19.45 kDa
1
0.8
0.6
0.4
0.2
0

Supplementary Figure S8. Heatmap depicting elution profiles of Ser-Leu and its targets identified in this work. Intensity was calculated relative to the maximum intensity of the protein subunit. Protein interactors were restricted to protein quantified in PROMIS experiment.

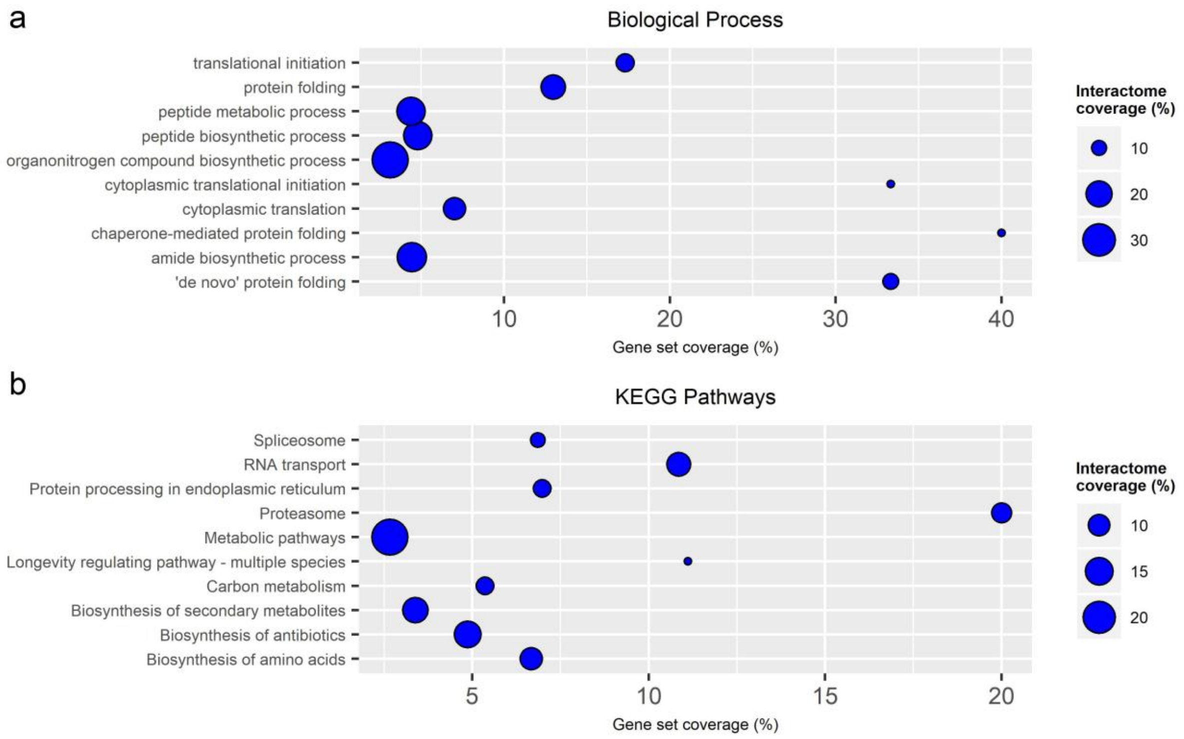

Supplementary Figure S9. Enrichment analysis of 77 proteins considered, as Ser-Leu interactome. a GO enrichment analysis. **b** KEGG pathways enrichment analysis. Top 10 most enriched processes are depicted (FDR < 0.05). Interactome coverage indicates relative coverage of the queried proteins. Gene set coverage indicates relative coverage of the respective enrichment category by queried proteins.

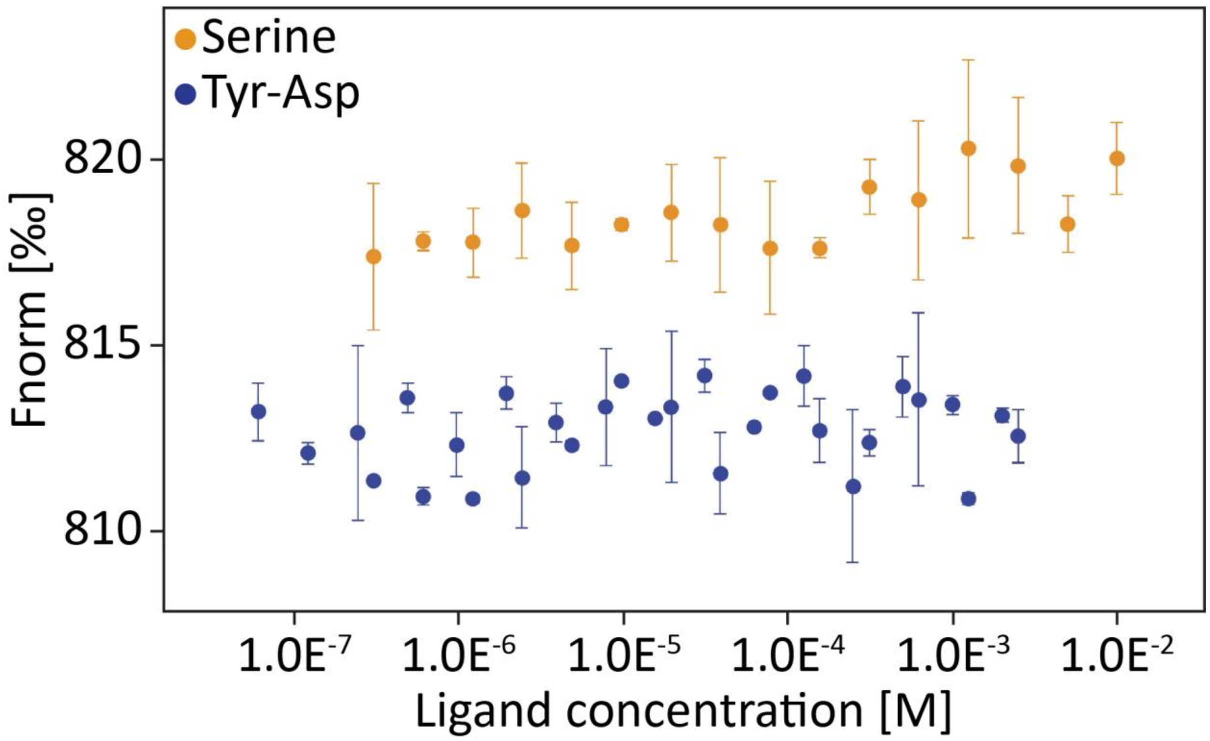

Supplementary Figure S10. *In vitro* validation of the interaction specificity between Pgk1 and Ser-Leu. MST analysis of the interaction between Pgk1 and Tyr-Asp, and Pgk1 and serine. Kd indicates dissociation constant. Error bars represent standard deviation. Binding was tested in three distinct replicates.

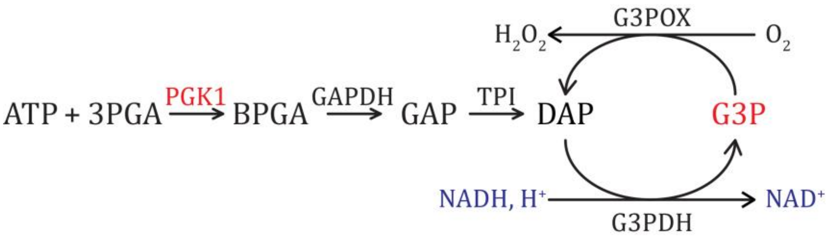

Supplementary Figure S11. Scheme showing components of the enzymatic assay used to measure Pgk1 activity in the presence and absence of Ser-Leu. 3PGA – 3-phosphoglycerate, BPGA – bisphosphoglycerate, GAPDH – glyceraldehyde-3-phosphate dehydrogenase, GAP – glyceraldehyde-3-phosphate, TPI – triose-phosphate isomerase, DAP – dihydroxyacetone phosphate, G3P – glycerol-3-phosphate, G3PDH – glycerol-3-phosphate dehydrogenase, G3POX – glycerol-3-phosphate oxidase. Pgk1 and the product of the reaction (G3P) are marked in red. The NADH is marked blue as its conversion was spectrophotometrically followed at 340 nm.

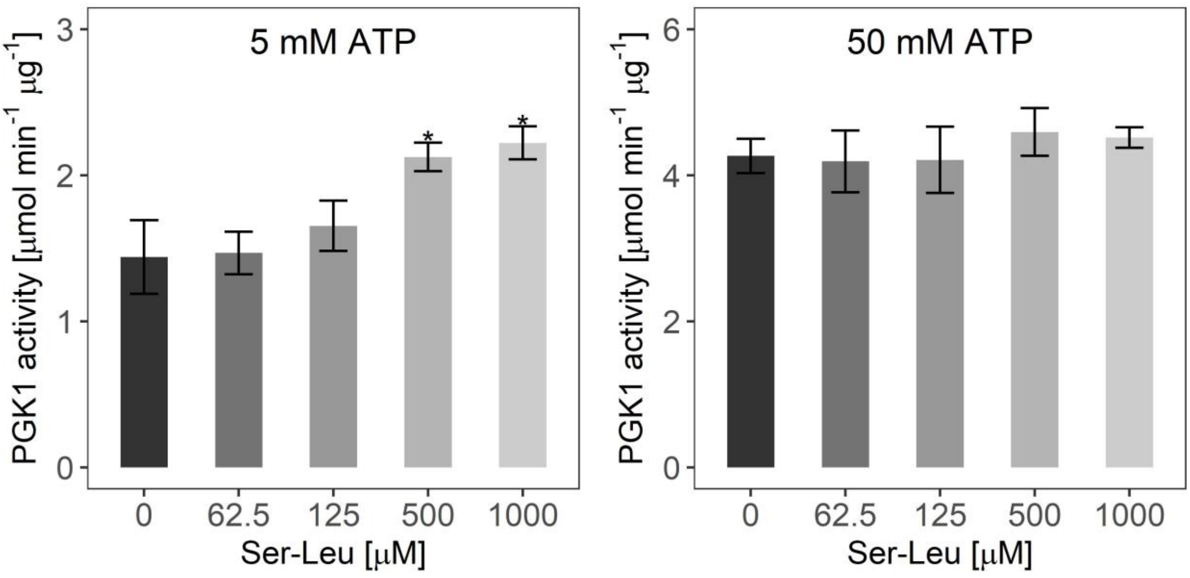

Supplementary Figure S12. Functional validation of the interaction between Ser-Leu and Pgk1. Ser-Leu significantly increases Pgk1 activity, when ATP concentration is low (1 and 5 mM). 50 mM ATP overcomes activating effect of Ser-Leu. Error bars represent standard deviation. Activity was tested in three independent replicates. Asterisks denote significant difference (non-paired, two-tailed t-test P-value < 0.05).

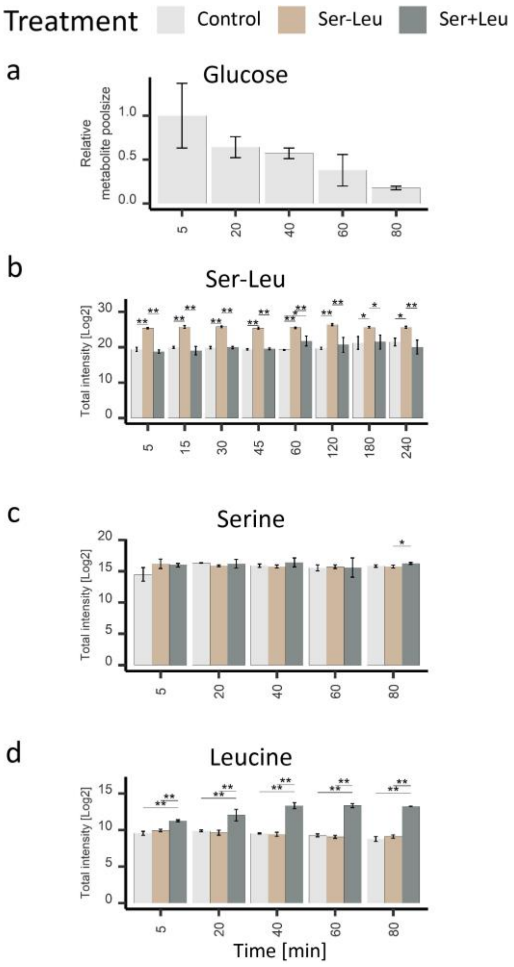

Supplementary Figure S13. YSBN2 response to Ser-Leu supplementation. LC- and GC-MS analysis of metabolomic changes caused upon supplementation with 100 µM Ser-Leu or mixture of 100 µM serine and 100 µM leucine. A) Analysis of glucose level in control samples. Shown are relative changes to time-point 0. Data represents the means ± SD, n=3. BCD) Presented are changes in metabolite levels (here described as total intensity). X-axis represents time [min] upon treatment. Data represents the means ± SD, n=3. Asterisks denote significant difference (Tukey's test, * *P*-value < 0.05, ** *P*-value < 0.01).

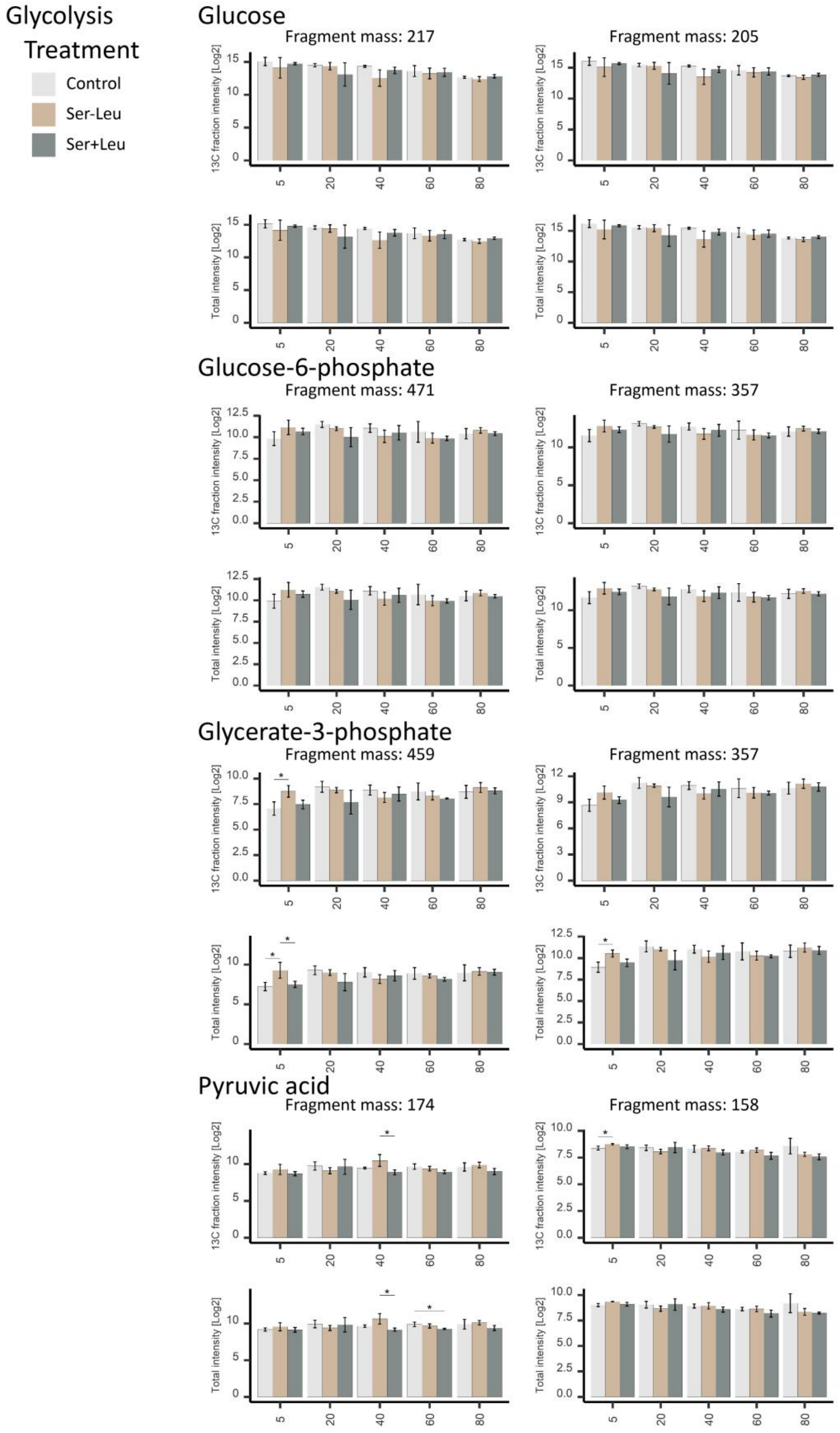

Supplementary Figure S14. YSBN2 response to Ser-Leu supplementation. LC- and GC-MS analysis of metabolomic changes caused upon supplementation with 100 µM Ser-Leu or mixture of 100 µM serine and 100 µM leucine. Presented are changes in metabolite levels (here described as total intensity) and redistribution of carbon isotope (enrichment level [%]

multiplied by metabolite level, here described as 13C fraction intensity) in yeast cells. ^{13}C enrichment in combination with metabolite levels provides information regarding the conversion rate of labelled substrate to the metabolite. X-axis represents time [min] upon treatment. Data represents the means ± SD, n=3. Asterisks denote significant difference (Tukey's test, * *P*-value < 0.05, ** *P*-value < 0.01).

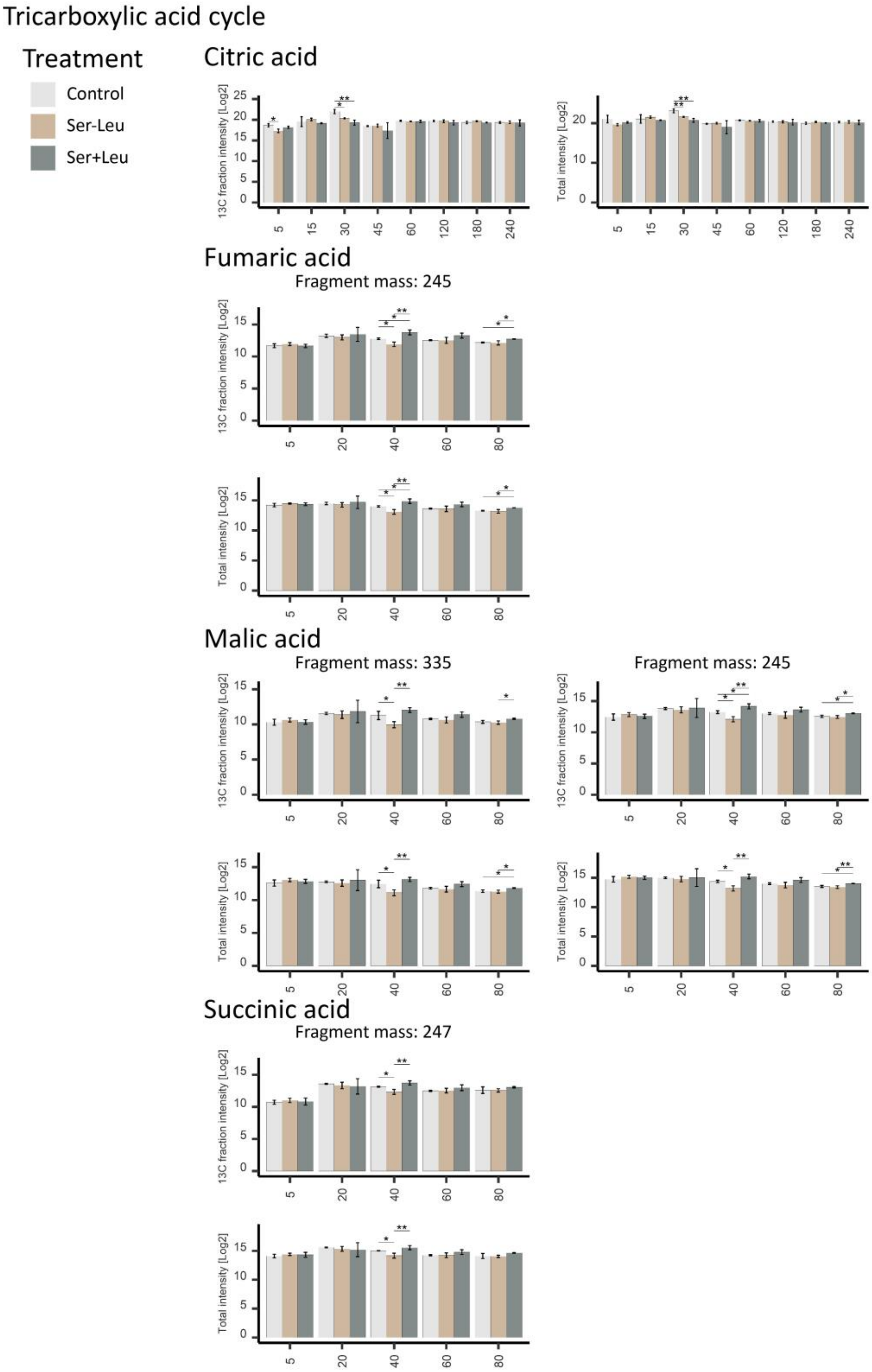

Supplementary Figure S15. YSBN2 response to Ser-Leu supplementation. LC- and GC-MS analysis of metabolomic changes caused upon supplementation with 100 µM Ser-Leu or mixture of 100 µM serine and 100 µM leucine. Presented are changes in metabolite levels (here described as total intensity) and redistribution of carbon isotope (enrichment level [%] multiplied by metabolite level, here described as 13C fraction intensity) in yeast cells. ^{13}C enrichment in combination with metabolite levels provides information regarding the conversion rate of labelled substrate to the metabolite. X-axis represents time [min] upon treatment. Data represents the means ± SD, n=3. Asterisks denote significant difference (Tukey's test, * *P*-value < 0.05, ** *P*-value < 0.01).

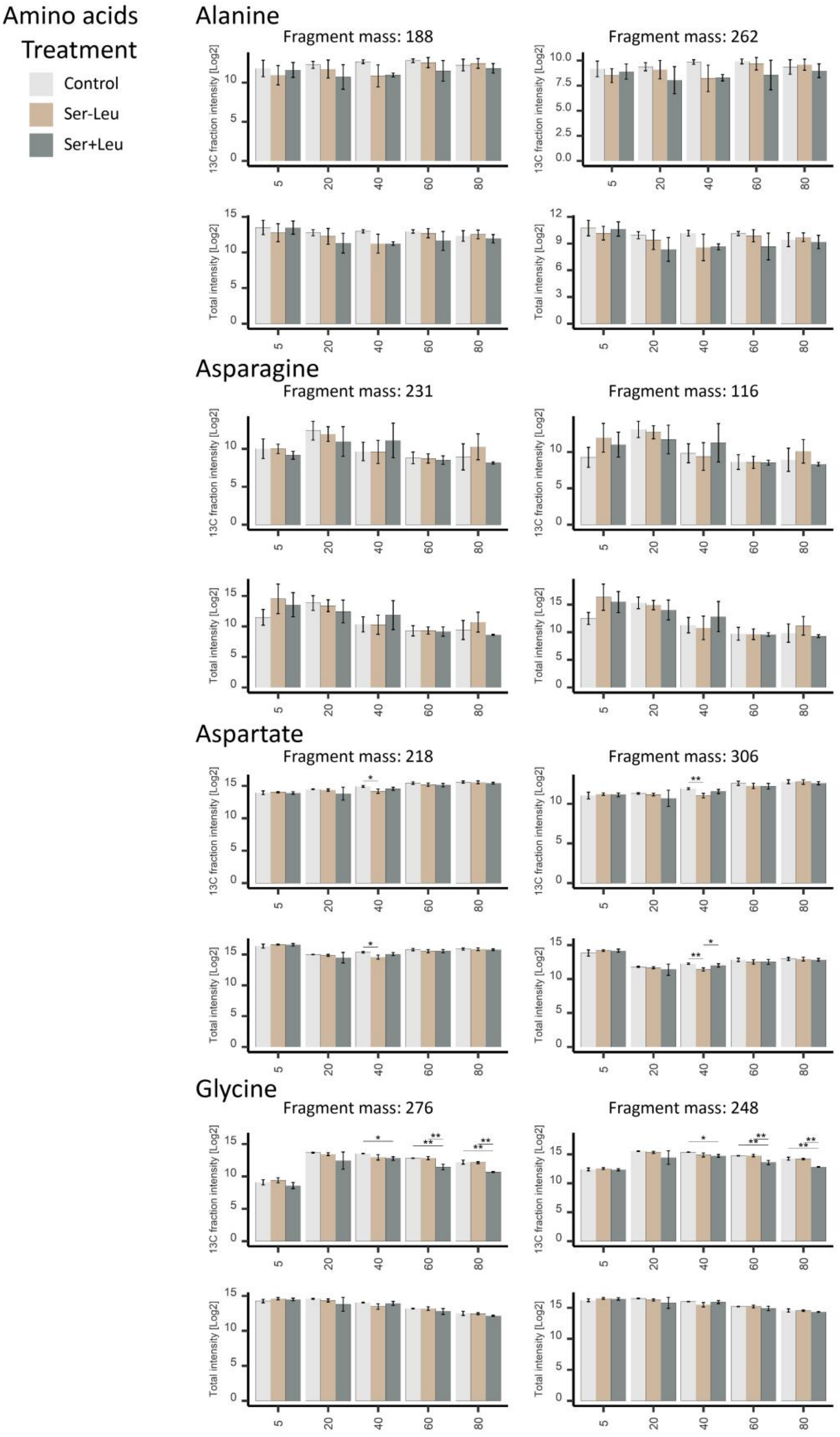

Supplementary Figure S16. YSBN2 response to Ser-Leu supplementation. LC- and GC-MS analysis of metabolomic changes caused upon supplementation with 100 µM Ser-Leu or mixture of 100 µM serine and 100 µM leucine. Presented are changes in metabolite levels (here described as total intensity) and redistribution of carbon isotope (enrichment level [%]

multiplied by metabolite level, here described as 13C fraction intensity) in yeast cells. ^{13}C enrichment in combination with metabolite levels provides information regarding the conversion rate of labelled substrate to the metabolite. X-axis represents time [min] upon treatment. Data represents the means ± SD, n=3. Asterisks denote significant difference (Tukey's test, * *P*-value < 0.05, ** *P*-value < 0.01).

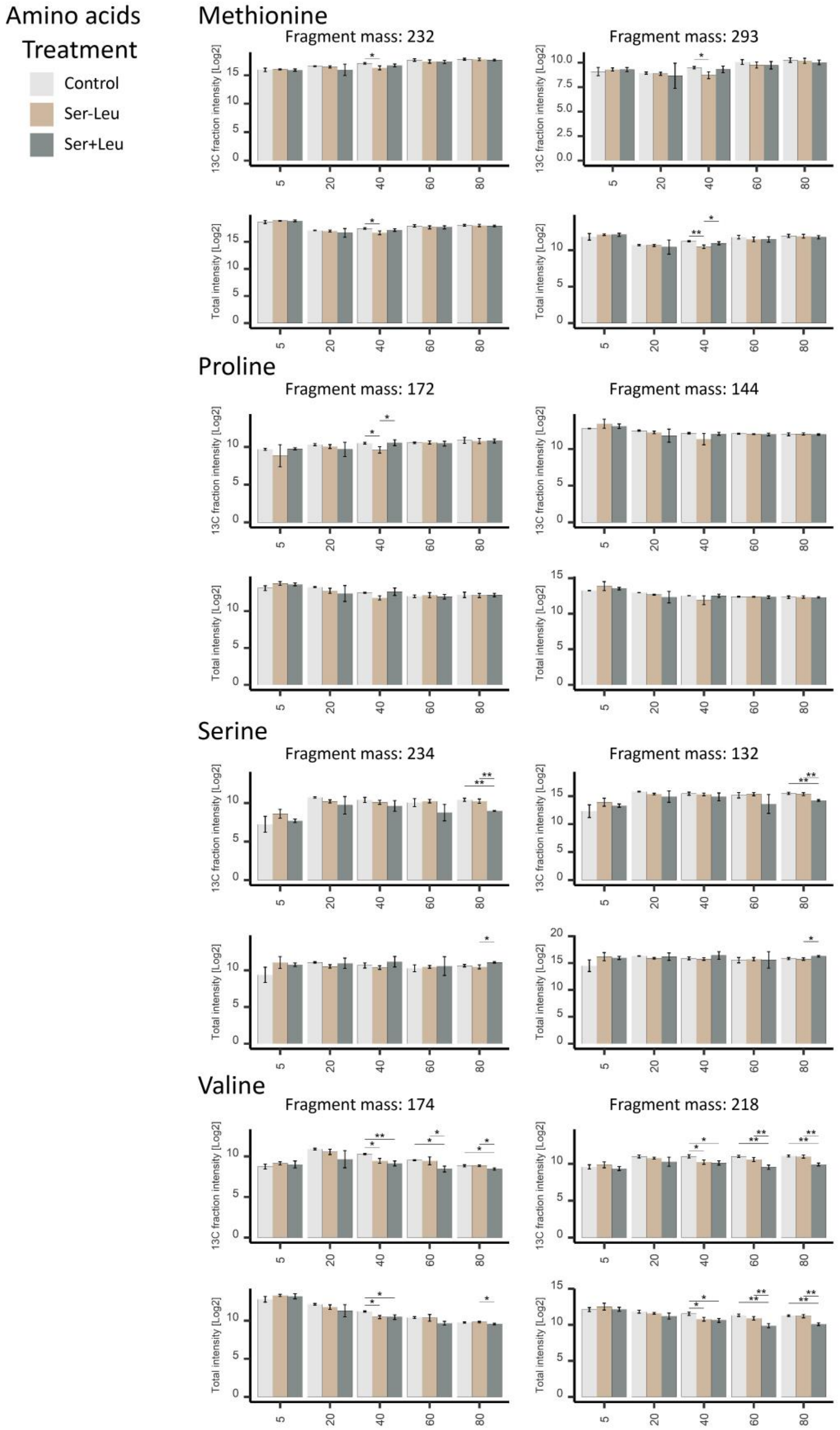

Supplementary Figure S17. YSBN2 response to Ser-Leu supplementation. LC- and GC-MS analysis of metabolomic changes caused upon supplementation with 100 µM Ser-Leu or mixture of 100 µM serine and 100 µM leucine. Presented are changes in metabolite levels (here described as total intensity) and redistribution of carbon isotope (enrichment level [%]

multiplied by metabolite level, here described as 13C fraction intensity) in yeast cells. ^{13}C enrichment in combination with metabolite levels provides information regarding the conversion rate of labelled substrate to the metabolite. X-axis represents time [min] upon treatment. Data represents the means ± SD, n=3. Asterisks denote significant difference (Tukey's test, * *P*-value < 0.05, ** *P*-value < 0.01).

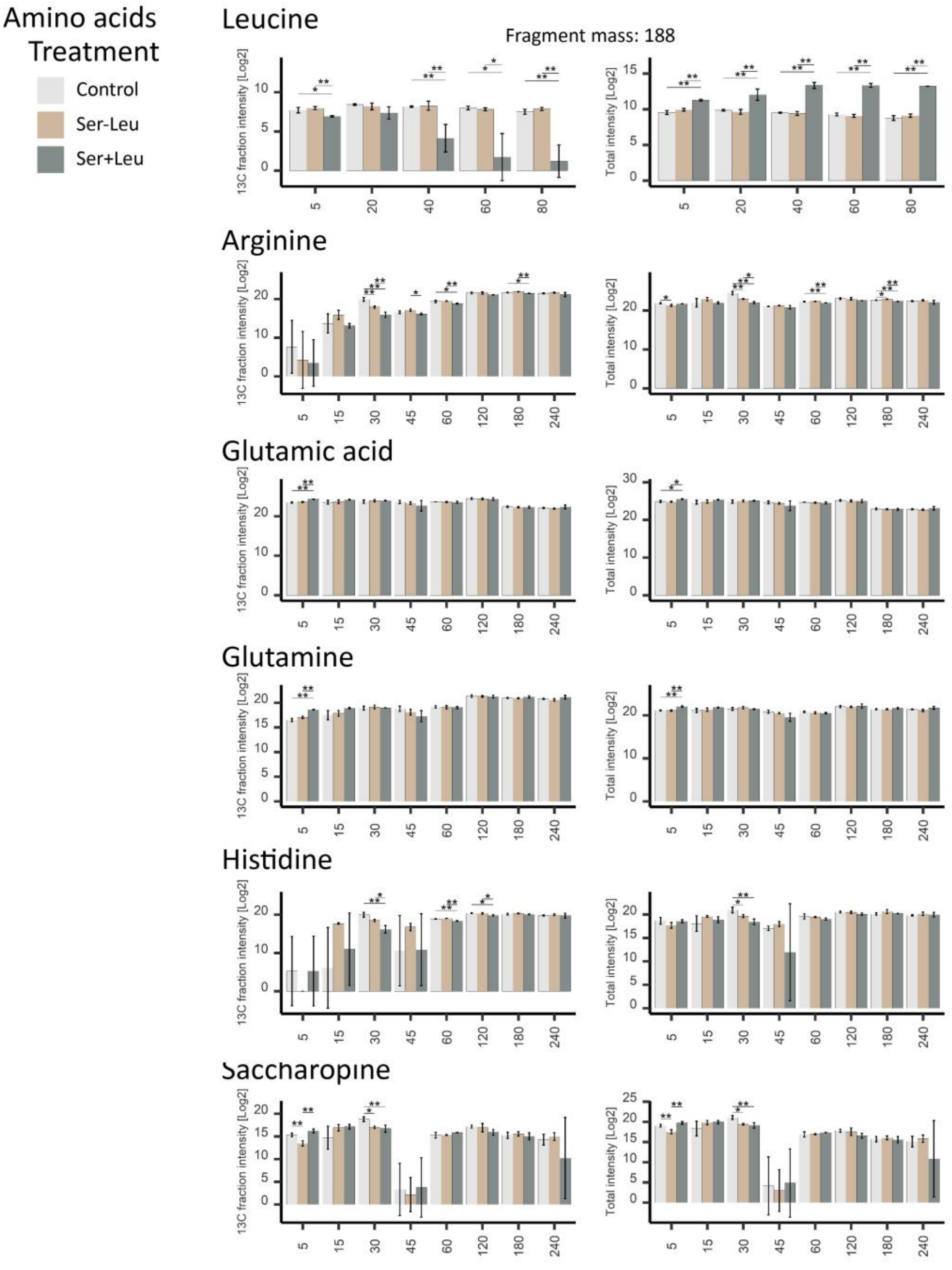

Supplementary Figure S18. YSBN2 response to Ser-Leu supplementation. LC- and GC-MS analysis of metabolomic changes caused upon supplementation with 100 µM Ser-Leu or mixture of 100 µM serine and 100 µM leucine. Presented are changes in metabolite levels (here described as total intensity) and redistribution of carbon isotope (enrichment level [%] multiplied by metabolite level, here described as 13C fraction intensity) in yeast cells. ^{13}C enrichment in combination with metabolite levels provides information regarding the conversion rate of labelled substrate to the metabolite. X-axis represents time [min] upon

treatment. Data represents the means ± SD, n=3. Asterisks denote significant difference (Tukey’s test, * *P*-value < 0.05, ** *P*-value < 0.01).

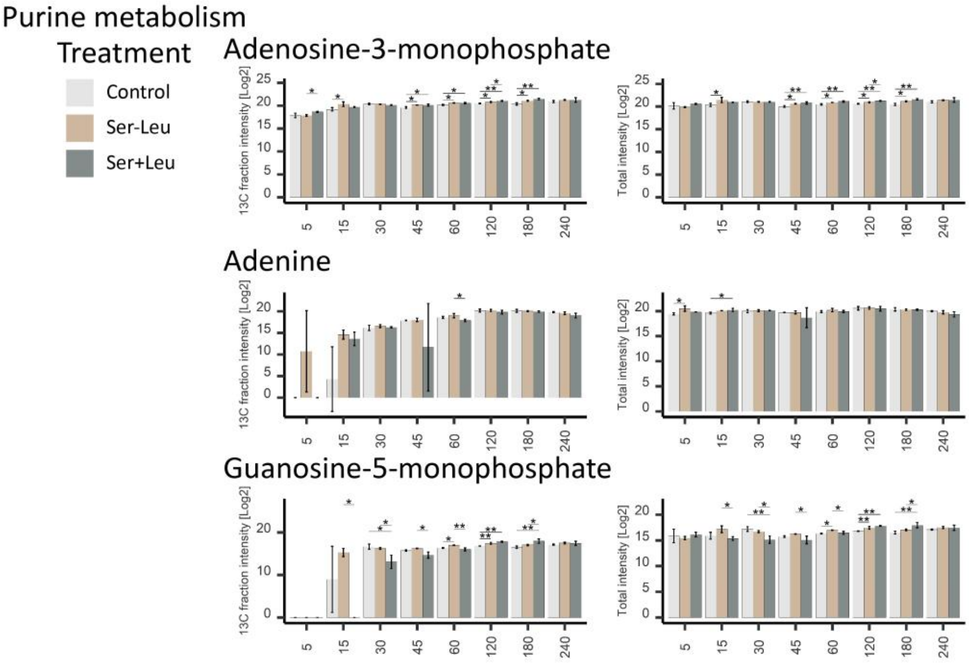

Supplementary Figure S19. YSBN2 response to Ser-Leu supplementation. LC- and GC-MS analysis of metabolomic changes caused upon supplementation with 100 µM Ser-Leu or mixture of 100 µM serine and 100 µM leucine. Presented are changes in metabolite levels (here described as total intensity) and redistribution of carbon isotope (enrichment level [%] multiplied by metabolite level, here described as 13C fraction intensity) in yeast cells. ^{13}C enrichment in combination with metabolite levels provides information regarding the conversion rate of labelled substrate to the metabolite. X-axis represents time [min] upon treatment. Data represents the means ± SD, n=3. Asterisks denote significant difference (Tukey’s test, * *P*-value < 0.05, ** *P*-value < 0.01).

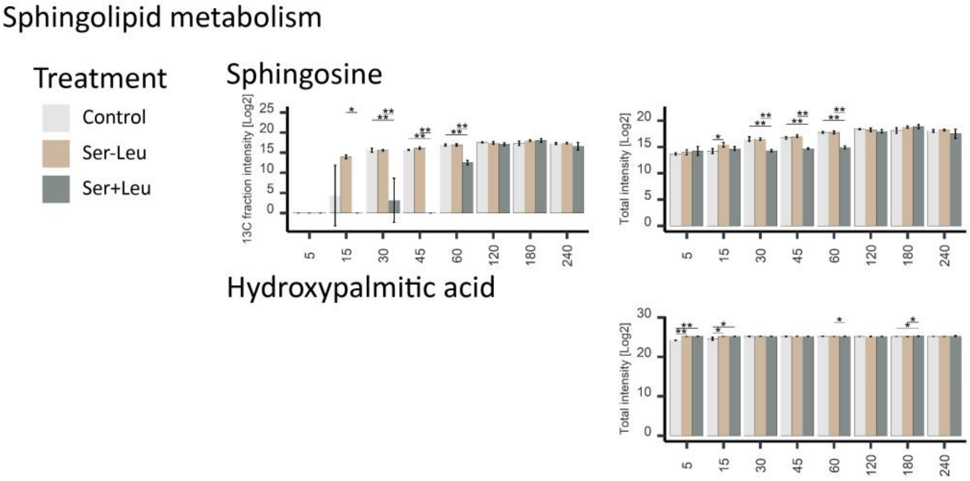

Supplementary Figure S20. YSBN2 response to Ser-Leu supplementation. LC- and GC-MS analysis of metabolomic changes caused upon supplementation with 100 µM Ser-Leu or mixture of 100 µM serine and 100 µM leucine. Presented are changes in metabolite levels

(here described as total intensity) and redistribution of carbon isotope (enrichment level [%] multiplied by metabolite level, here described as 13C fraction intensity) in yeast cells. ^{13}C enrichment in combination with metabolite levels provides information regarding the conversion rate of labelled substrate to the metabolite. X-axis represents time [min] upon treatment. Data represents the means ± SD, n=3. Asterisks denote significant difference (Tukey's test, * *P*-value < 0.05, ** *P*-value < 0.01).

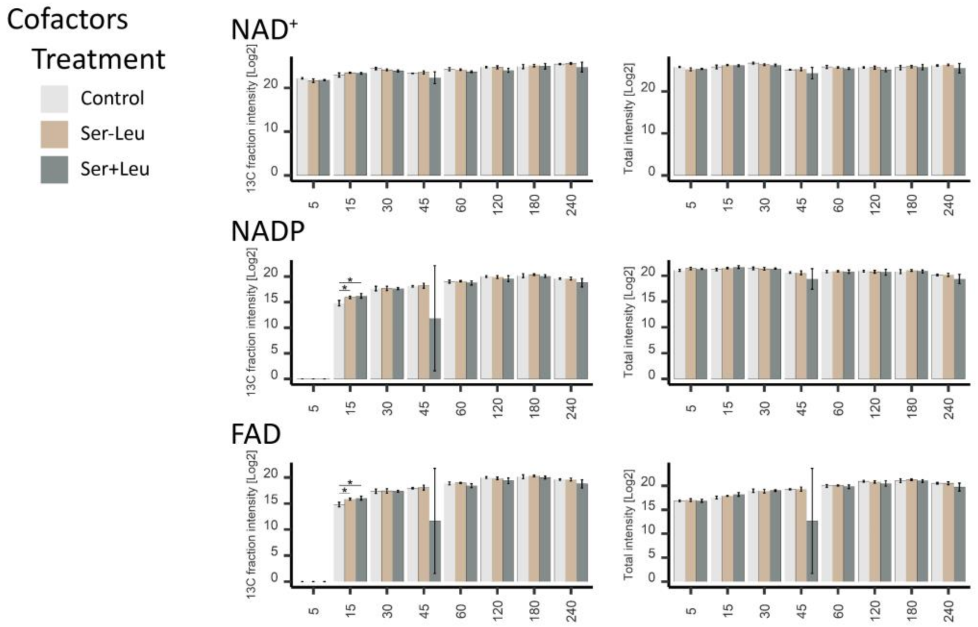

Supplementary Figure S21. YSBN2 response to Ser-Leu supplementation. LC- and GC-MS analysis of metabolomic changes caused upon supplementation with 100 µM Ser-Leu or mixture of 100 µM serine and 100 µM leucine. Presented are changes in metabolite levels (here described as total intensity) and redistribution of carbon isotope (enrichment level [%] multiplied by metabolite level, here described as 13C fraction intensity) in yeast cells. ^{13}C enrichment in combination with metabolite levels provides information regarding the conversion rate of labelled substrate to the metabolite. X-axis represents time [min] upon treatment. Data represents the means ± SD, n=3. Asterisks denote significant difference (Tukey's test, * *P*-value < 0.05, ** *P*-value < 0.01).

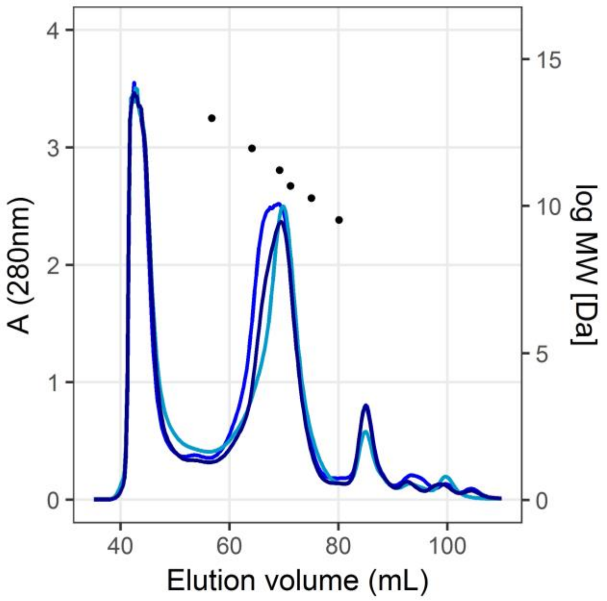

Supplementary Figure S22. Chromatograms of the absorption at 280 nm of three independent replicas. The approximate protein molecular weight distribution as determined using protein standards is plotted as black dots.

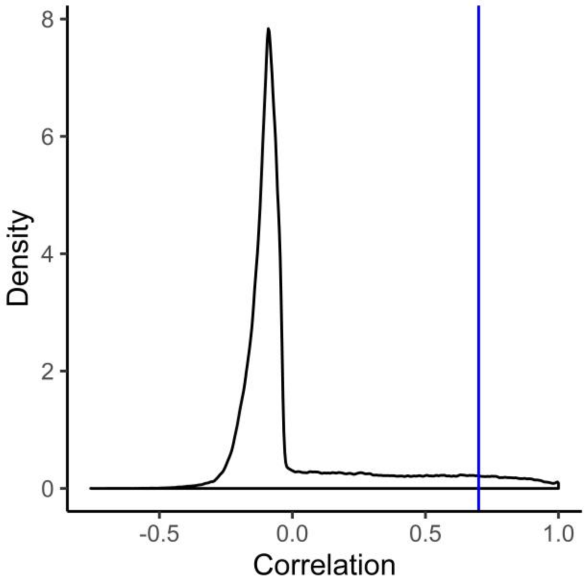

Supplementary Figure S23. Overall distribution of correlation coefficients for all peak pairs. Blue line indicates the cut-off selected for predicting interactions.

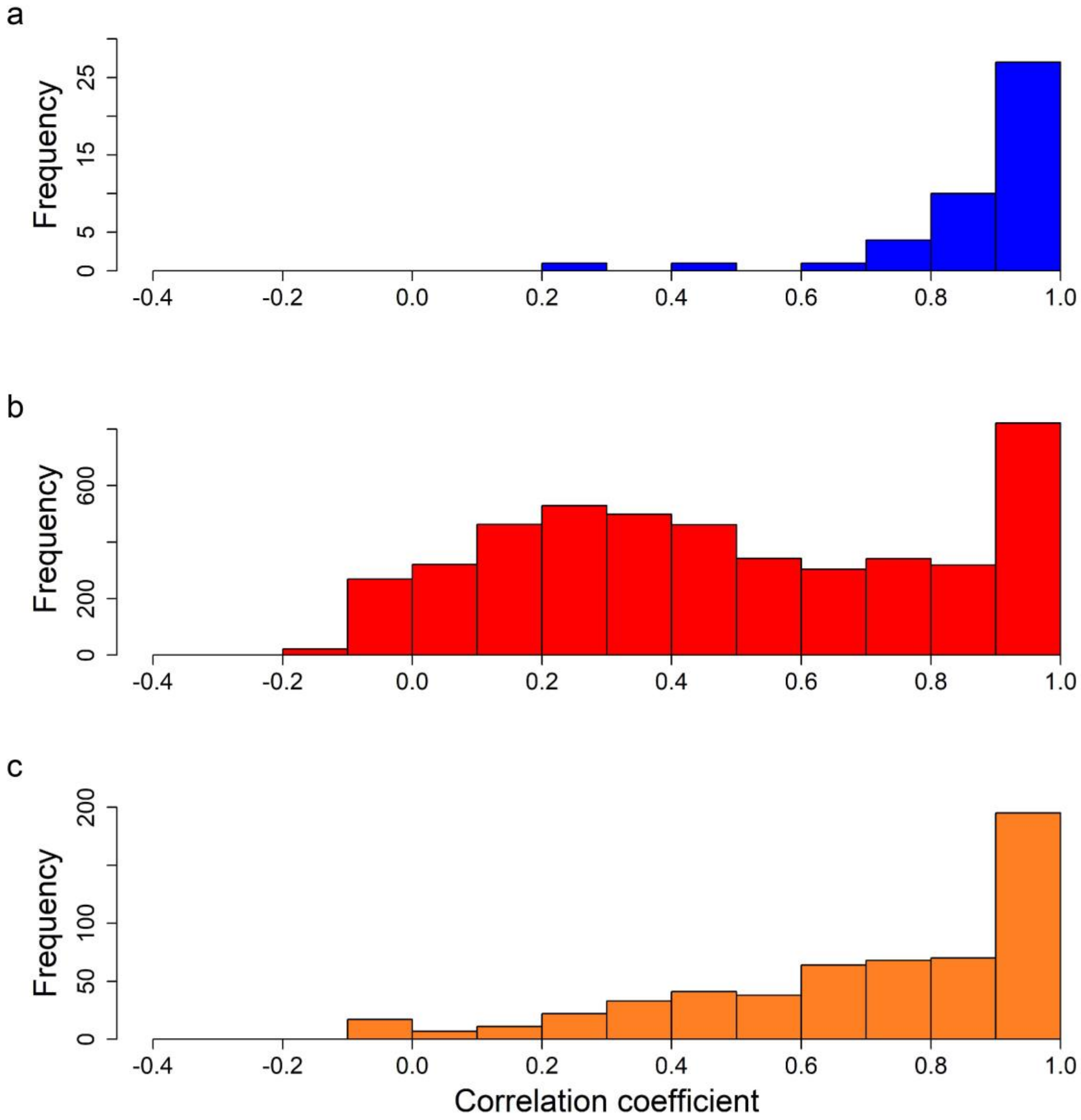

Supplementary Figure S24. Distribution of the Pearson correlation in PROMIS dataset. PCC was calculated between elution profiles of annotated metabolites (top panel) and non-annotated metabolites measured in positive (middle panel) and negative (bottom panel) mode shows enrichment of compounds having reproducible elution profile (PCC >= 0.9) between at least two replica.

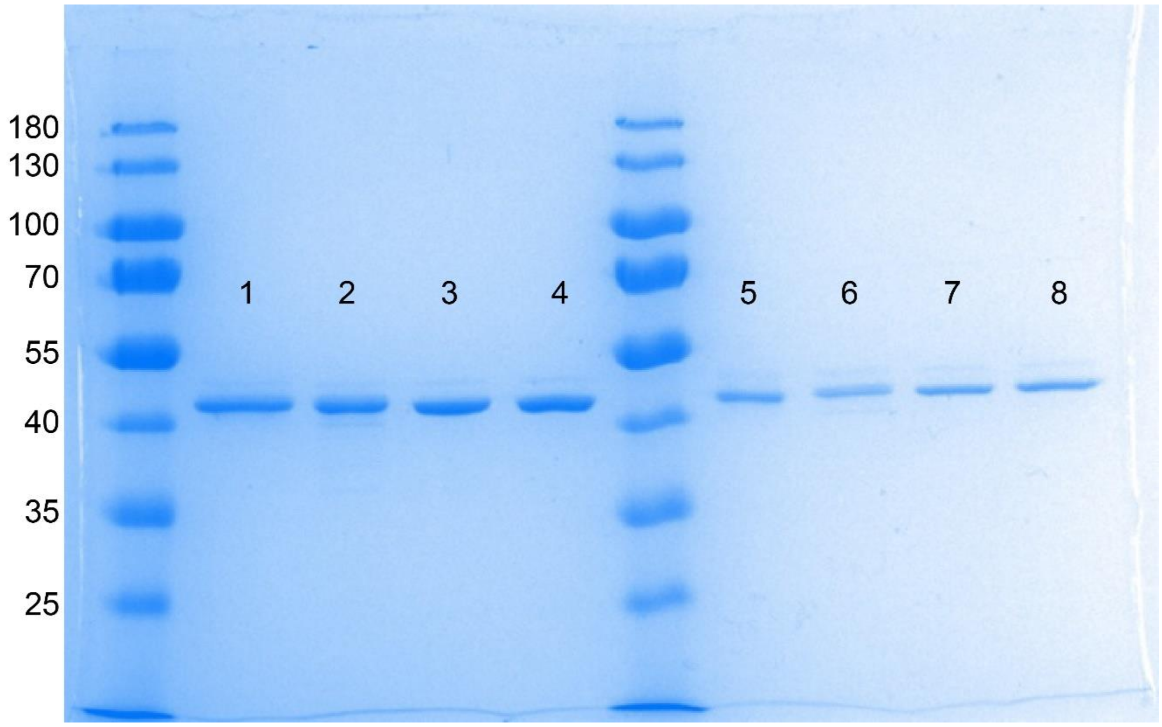

Supplementary Figure S25. SDS-PAGE analysis of Pgk1 purity. 1-4. Commercially available Pgk1 (Sigma) **5-8.** Pgk1 overexpressed and purified using commercially available strain (c.f. materials and methods). Equal volume of each sample was loaded on the protein gel.

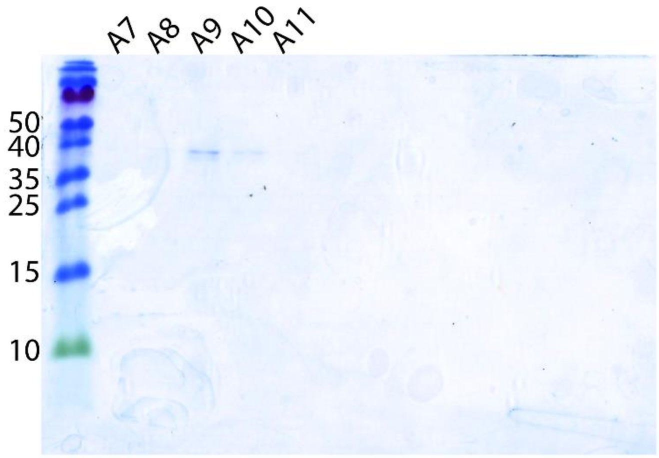

Supplementary Figure S26. SDS-PAGE analysis of Pnp1 purity after two-step purification. Equal volume of each fraction was loaded on the protein gel.

References

1 Canelas, A. B. *et al.* Integrated multilaboratory systems biology reveals differences in protein metabolism between two reference yeast strains. *Nature communications* **1**, 145 (2010).

2 Gelperin, D. M. *et al.* Biochemical and genetic analysis of the yeast proteome with a movable ORF collection. *Genes & development* **19**, 2816-2826 (2005).

3 Cox, J. & Mann, M. MaxQuant enables high peptide identification rates, individualized ppb-range mass accuracies and proteome-wide protein quantification. *Nature biotechnology* **26**, 1367-1372 (2008).

4 Cox, J. *et al.* Andromeda: a peptide search engine integrated into the MaxQuant environment. *Journal of proteome research* **10**, 1794-1805 (2011).

5 R Core Team. R: A language and environment for statistical computing. *R Foundation for Statistical Computing, Vienna, Austria. URL* https://www.R-project.org/ (2018).

6 RStudio Team. RStudio: Integrated Development Environment for R. *RStudio, PBC, Boston, MA URL* http://www.rstudio.com/ (2020).

7 Rappsilber, J., Ishihama, Y. & Mann, M. Stop and go extraction tips for matrix-assisted laser desorption/ionization, nanoelectrospray, and LC/MS sample pretreatment in proteomics. *Analytical chemistry* **75**, 663-670 (2003).

8 Veyel, D. *et al.* System-wide detection of protein-small molecule complexes suggests extensive metabolite regulation in plants. *Scientific Reports* **7**, doi:10.1038/srep42387 (2017).

9 Sokolowska, E. M., Schlossarek, D., Luzarowski, M. & Skirycz, A. PROMIS: Global Analysis of PROtein-Metabolite Interactions. *Current protocols in plant biology* **4**, e20101 (2019).

10 Balakrishnan, R. *et al.* YeastMine—an integrated data warehouse for Saccharomyces cerevisiae data as a multipurpose tool-kit. **2012**, doi:https://dx.doi.org/10.1093%2Fdatabase%2Fbar062 (2012).

11 Cattaneo, G. *et al.* Development, validation and application of a 96-well enzymatic assay based on LC-ESI-MS/MS quantification for the screening of selective inhibitors against Mycobacterium tuberculosis purine nucleoside phosphorylase. *Analytica chimica acta* **943**, 89-97 (2016).

12 Giavalisco, P. *et al.* Elemental formula annotation of polar and lipophilic metabolites using 13C, 15N and 34S isotope labelling, in combination with high-resolution mass spectrometry. *The Plant Journal* **68**, 364-376 (2011).

13 Savitski, M. M. *et al.* Tracking cancer drugs in living cells by thermal profiling of the proteome. *Science* **346**, 1255784 (2014).

14 Childs, D. *et al.* Non-Parametric Analysis of Thermal Proteome Profiles Reveals Novel Drug-Binding Proteins. *bioRxiv*, 373845 (2018).

15 Steinhauser, M.-C. *et al.* Enzyme activity profiles during fruit development in tomato cultivars and Solanum pennellii. *Plant Physiology* **153**, 80-98 (2010).

16 Gibon, Y., Vigeolas, H., Tiessen, A., Geigenberger, P. & Stitt, M. Sensitive and high throughput metabolite assays for inorganic pyrophosphate, ADPGlc, nucleotide phosphates, and glycolytic intermediates based on a novel enzymic cycling system. *The Plant Journal* **30**, 221-235 (2002).

17 Jorgensen, P., Nishikawa, J. L., Breitkreutz, B.-J. & Tyers, M. Systematic identification of pathways that couple cell growth and division in yeast. *Science* **297**, 395-400 (2002).

18 Burtner, C. R., Murakami, C. J., Kennedy, B. K. & Kaeberlein, M. A molecular mechanism of chronological aging in yeast. *Cell cycle* **8**, 1256-1270 (2009).

19 Kanshin, E., Kubiniok, P., Thattikota, Y., D'Amours, D. & Thibault, P. Phosphoproteome dynamics of Saccharomyces cerevisiae under heat shock and cold stress. *Molecular systems biology* **11** (2015).

20 Erban, A., Schauer, N., Fernie, A. R. & Kopka, J. Nonsupervised construction and application of mass spectral and retention time index libraries from time-of-flight gas chromatography-mass spectrometry metabolite profiles. *Metabolomics*, 19-38 (2007).
21 Dethloff, F. *et al.* Profiling methods to identify cold-regulated primary metabolites using gas chromatography coupled to mass spectrometry. *In Plant Cold Acclimation*, 171-197 (2014).
22 Wagner, C., Sefkow, M. & Kopka, J. Construction and application of a mass spectral and retention time index database generated from plant GC/EI-TOF-MS metabolite profiles. *Phytochemistry* **62**, 887-900 (2003).
23 Luedemann, A., Strassburg, K., Erban, A. & Kopka, J. TagFinder for the quantitative analysis of gas chromatography—mass spectrometry (GC-MS)-based metabolite profiling experiments. *Bioinformatics* **24**, 732-737 (2008).
24 Allwood, J. W. *et al.* Inter-laboratory reproducibility of fast gas chromatography–electron impact–time of flight mass spectrometry (GC–EI–TOF/MS) based plant metabolomics. *Metabolomics* **5**, 479-496 (2009).
25 Kopka, J. *et al.* GMD@ CSB. DB: the Golm metabolome database. *Bioinformatics* **21**, 1635-1638 (2005).
26 Hummel, J., Strehmel, N., Selbig, J., Walther, D. & Kopka, J. Decision tree supported substructure prediction of metabolites from GC-MS profiles. *Metabolomics* **6**, 322-333 (2010).
27 Strehmel, N., Hummel, J., Erban, A., Strassburg, K. & Kopka, J. Retention index thresholds for compound matching in GC–MS metabolite profiling. *Journal of Chromatography B* **871**, 182-190 (2008).
28 Huege, J., Goetze, J., Dethloff, F., Junker, B. & Kopka, J. in *Plant Chemical Genomics: Methods and Protocols* (eds Glenn R. Hicks & Stéphanie Robert) 213-223 (Humana Press, 2014).
29 Huege, J. *et al.* Modulation of the major paths of carbon in photorespiratory mutants of Synechocystis. *PLoS One* **6** (2011).
30 Huege, J. *et al.* GC-EI-TOF-MS analysis of in vivo carbon-partitioning into soluble metabolite pools of higher plants by monitoring isotope dilution after 13CO2 labelling. *Phytochemistry* **68**, 2258-2272 (2007).
