# Supplementary material for: Global mapping of protein–metabolite interactions in Saccharomyces cerevisiae reveals that Ser-Leu dipeptide regulates phosphoglycerate kinase activity
Source: Commun Biol. 2021 Feb 10;4:181. doi: 10.1038/s42003-021-01684-3 (PMC7876005; doi:10.1038/s42003-021-01684-3)
Supplement: Supplementary file 3 — Description of Additional Supplementary Files [file 42003_2021_1684_MOESM3_ESM.pdf]

Description of Additional Supplementary Files

File name: Supplementary Data 1

Description:

Data S1. Small molecules separating together with protein complexes, and thus classified as protein bound. Column names indicate theoretical molecular weight calculated based on the reference proteins.
Data S2. Small molecules that could be annotated to a specific compound using chemical standards. Column names indicate theoretical molecular weight calculated based on the reference proteins.
Data S3. 3982 proteins identified using LC-MS in protein containing fractions. For filtering criteria, see experimental procedures.
Data S4. PANTHER db output data. 3982 proteins identified in SEC experiment were queried against PANTHER database to perform overrepresentation analysis.
Data S5. Deconvoluted elution profiles of unknown metabolites present in protein containing fraction and thus classified as protein bound. Column names indicate theoretical molecular weight calculated based on the reference proteins.
Data S6. Deconvoluted elution profiles of annotated metabolites present in protein containing fraction and thus classified as protein bound. Column names indicate theoretical molecular weight calculated based on the reference proteins.
Data S7. Deconvoluted elution profiles of identified proteins. Column names indicate theoretical molecular weight calculated based on the reference proteins.
Data S8. A list of proteins in a complex was determined by calculating oligomeric state ratio (OSR). OSR is the ratio of the apparent mass of a protein, calculated using reference proteins, to the theoretical monomeric mass of a protein.
Data S9. List of 87 PMI obtained by restricting STITCH database to proteins and metabolites quantified in PROMIS experiment. Experimentally confirmed interactions passing 800 confidence threshold were considered as known protein – metabolite interactions. Negative correlation values were replaced by 0. Maximum correlation coefficient is reported between elution profiles.
Data S10. True and false positive rates for the different correlation coefficient cut-off. Presented are two approaches for determining False Positive Rate. For more details, please see Methods.
Data S11. 225 protein - metabolite interactions, engaging 22 unique metabolites for which this study provide experimental validation.
Data S12. 162 significantly enriched proteins in AP eluates coming from the Ser-Leu_N and Ser-Leu-C beads, constituting putative Ser-Leu targets.
Data S13. 94 putative targets of Ser-Leu identified using TPP using NPARC method (p-value ≤ 0.05).
Data S14. All mass features quantified using LC-MS in positive mode. Rep_x indicates number of replica. Non-prot indicates negative control without proteins.
Data S15. All mass features quantified using LC-MS in negative mode. Rep_x indicates number of replica. Non-prot indicates negative control without proteins.
Data S16. MaxQuant output parameters.txt file. It contains parameters used for processing RAW chromatograms.
Data S17. Similarity between deconvoluted elution profiles of annotated metabolites and proteins was determined by calculated Pearson correlation coefficient. Data represents correlation matrix.
Data S18. Mass features quantified using LC/MS in PNP1 enzymatic assay. Cluster_0847 was annotated as hypoxanthine using reference compound library, allowing 5 ppm mass deviation and 0.1 min RT deviation. Data created based on the output file of Refiner MS, which was used for processing of raw chromatograms.
Data S19. MaxQuant output parameters.txt file. It contains parameters used for processing RAW chromatograms of AP experiment.

Data S20. 913 proteins identified using LC-MS in AP experiment. For filtering criteria, please see experimental procedures.
Data S21. Results of two-way ANOVA followed by Tukey's test performed on LFQ intensities acquired for proteins captured in AP experiment with Ser-Leu immobilized beads.
Data S22. 162 significantly enriched proteins in corresponding eluates from agarose beads in comparison to empty beads control (ANOVA $p < 0.05$, Tukey's $p < 0.05$).
Data S23. MaxQuant output parameters.txt file. It contains parameters used for processing RAW chromatograms of TPP experiment.
Data S24. 1798 proteins identified using LC-MS in TPP experiment. For filtering criteria, please see experimental procedures.
Data S25. 1582 proteins processed using TPP package. For more details see Childs et al., 2018
Data S26. In vitro validation of PGK1:Ser-Leu interaction using microscale thermophoresis.
Data S27. In vitro validation of PGK1:ATP interaction in presence and absence of 4 mM Ser-Leu using microscale thermophoresis.
Data S28. Results of enymatic activity assay. Tested was activity of Pgk1 [μmol μg^{-1} min^{-1}] in presence of different concentrations of Ser-Leu at different concentrations of substrate (ATP) used in the assay.
Data S29. Estimation of Ser-Leu level in yeast.
Data S30. Dipeptide and amino acid accumulation during growth on glucose at optimal conditions.
Data S31. Effect of Ser-Leu supplementation on yeast growth. Shown is optical density at 600nm.
Data S32. Changes in metabolism upon Ser-Leu supplementation (13C-isotope-labeling experiment) followed by GC-MS analysis.
Data S33. Changes in metabolism upon Ser-Leu supplementation (13C-isotope-labeling experiment) followed by LC-MS analysis.
